# Two cell line models to study multiorganic metastasis and immunotherapy in lung squamous cell carcinoma

**DOI:** 10.1242/dmm.049137

**Published:** 2022-01-31

**Authors:** Karmele Valencia, Cristina Sainz, Cristina Bértolo, Gabriel de Biurrun, Jackeline Agorreta, Arantza Azpilikueta, Marta Larrayoz, Graziella Bosco, Carolina Zandueta, Miriam Redrado, Esther Redín, Francisco Exposito, Diego Serrano, Mirari Echepare, Daniel Ajona, Ignacio Melero, Ruben Pio, Roman Thomas, Alfonso Calvo, Luis M. Montuenga

**Affiliations:** 1 Program in Solid Tumors, CIMA-University of Navarra, 31008 Pamplona, Spain; 2 Consorcio de Investigación Biomédica en Red de Cáncer (CIBERONC), 28029 Madrid, Spain; 3 Navarra Health Research Institute (IDISNA), 31008 Pamplona, Spain; 4 Department of Biochemistry and Genetics, School of Sciences, University of Navarra, 31009 Pamplona, Spain; 5 Department of Environmental Biology School of Sciences, University of Navarra, 31009 Pamplona, Spain; 6 Department of Health Sciences, Biochemistry Area, Public University of Navarra, 31008 Pamplona, Spain; 7 Program of Immunology and Immunotherapy, CIMA-University of Navarra, 31008 Pamplona, Spain; 8 Department of Pathology, Anatomy and Physiology, School of Medicine, University of Navarra, 31009 Pamplona, Spain; 9 Department of Translational Genomics, Medical Faculty, University of Cologne, 50931 Cologne, Germany; 10 Department of Oncology, Clínica Universidad de Navarra, 31008 Pamplona, Spain; 11 Department of Pathology, University Hospital Cologne, 50937 Cologne, Germany; 12 German Cancer Research Center, German Cancer Consortium (DKTK), 69120 Heidelberg, Germany

**Keywords:** Lung cancer, Squamous, NTCU-mouse model, Syngeneic cell lines, RNASeq, Immunotherapy

## Abstract

There is a paucity of adequate mouse models and cell lines available to study lung squamous cell carcinoma (LUSC). We have generated and characterized two models of phenotypically different transplantable LUSC cell lines, i.e. UN-SCC679 and UN-SCC680, derived from A/J mice that had been chemically induced with N-nitroso-tris-chloroethylurea (NTCU). Furthermore, we genetically characterized and compared both LUSC cell lines by performing whole-exome and RNA sequencing. These experiments revealed similar genetic and transcriptomic patterns that may correspond to the classic LUSC human subtype. In addition, we compared the immune landscape generated by both tumor cells lines *in vivo* and assessed their response to immune checkpoint inhibition. The differences between the two cell lines are a good model for the remarkable heterogeneity of human squamous cell carcinoma. Study of the metastatic potential of these models revealed that both cell lines represent the organotropism of LUSC in humans, i.e. affinity to the brain, bones, liver and adrenal glands. In summary, we have generated valuable cell line tools for LUSC research, which recapitulates the complexity of the human disease.

## Abstract (precis)

**Summary:** Generation and characterization of two phenotypically different and transplantable mouse model lung squamous cell carcinoma cell lines – UN-SCC679 and UN-SCC680 – to be used in different research scenarios, including immunotherapy.

## INTRODUCTION

Lung cancer is the leading cause of cancer-related death worldwide (Ferlay et al., 2019). The incidence and mortality associated with lung cancer is a major public health challenge in advanced societies (Siegel et al., 2020). It is expected that, by 2035, less-developed regions will face an increase of new cancer cases by 144%, compared to 54% in more-developed regions (Pilleron et al., 2019). Lung cancer represents ∼12-13% of all new diagnosed cases and presents the highest overall mortality.

Of all lung cancers ∼20-30% are classified as lung squamous cell carcinoma (LUSC; also known as SCC or SqCC of the lung, or epidermoid carcinoma). There are two types of lung cancer, i.e. small lung cell cancer (SCLC) and non-small cell lung cancer (NSCLC), and LUSC is more strongly associated with tobacco smoking than any other subtype of NSCLC. Age, exposure to second-hand smoke, asbestos, mineral and metal dust, and radon constitute other risk factors for LUSC.

Basal stratified squamous lung epithelial cells are the most likely origin for LUSC (Ferone et al., 2020). Within LUSC, different histological subtypes ranging from the most-differentiated keratinized subtype to a mainly undifferentiated subtype without apparent signs of keratinization can be distinguished. For an accurate pathological diagnosis, the use of immunohistochemical markers is mandatory. LUSC is characterized for being positive for cytokeratins 5 and 6 (CKs5/6; officially known as KRT5 and KRT6, respectively) as well as for tumor protein p63 (officially known as TP63) and its isoform p40, and for being negative for thyroid transcription factor-1 (TTF1, officially known as TITF1), the pathognomonic immunohistochemical marker of the NSCLC subtype lung adenocarcinoma (LUAD) (Goldstraw et al., 2016). More frequently, LUSC occurs in the central airways or in higher order bronchi but, as well as to lung-related lymph nodes, it can spread to multiple organs, including brain, bones, adrenal glands and liver (Milovanovic et al., 2017).

LUSC is a less-studied NSCLC subtype than LUAD. Different factors have contributed this fact. First LUSC biology is more challenging than that of LUAD and, on average, human LUSCs have more mutations per megabase than LUAD (Bailey et al., 2018). Thus, LUSC may have higher genetic and phenotypic heterogeneity might be increased in LUSC. The lack of evident oncogenic driver mutations within LUSC is also a hurdle; instead, it seems to be driven by copy number change and/or mutations of tumor suppressors. In fact, although there have been some attempts to target recurrent genetic alterations, targeted therapies were demonstrated to be poorly effective options for LUSC up to now (Rekhtman et al., 2012). Second, the scarcity of preclinical models makes disease analysis and research more challenging. Thus, LUSC animal models to date have mostly been developed in mice after intratracheal instillation of chemicals, such as benzo-[a]-pyrene (BaP) or 3-methylholanthrene (3-MCA) (Whitmire et al., 1981; Yoshimoto et al., 1977), or after topical application of N-nitroso-methyl-bis-chloromethyl urea or N-nitroso-tris-chloroethylurea (NTCU) (Wang et al., 2004). To our knowledge only one cell line has – >40 years ago – been derived from one of these mouse models; it was used for a number of experiments over the years, although its full genomic and phenotypic characteristics have not yet been described (Kaneko and LePage TKS, 1980). There are also a limited number of genetically engineered mouse models (GEMMs) (Ferone et al., 2020). In the era of immunotherapy, the use of well-characterized murine LUSC cell lines that can be transplanted in syngeneic mice is highly convenient to test novel therapies. Such approach is more-complex in chemically induced and GEMMs because of their late tumor onset and slower tumor growth.

In this present work, we performed exhaustive molecular and functional characterization, and comparison of two novel LUSC research cell line models. These models consist of two A/J mouse-derived syngeneic cell lines obtained by using NTCU-induced LUSCs. These cell lines might become robust tools for the study of squamous cell lung cancer in a reliable and reproducible manner, and when testing novel antimetastatic and immunotherapy agents. In fact, one of the two cell lines (UN-SCC680) has successfully been used by our group to evaluate a strategy of combined immunotherapy (Azpilikueta et al., 2016). Here, we provide essential and comparative information that will allow the use of these models in molecularly defined experimental settings

## RESULTS

### Generation of LUSC cell lines

**Fig. 1.**
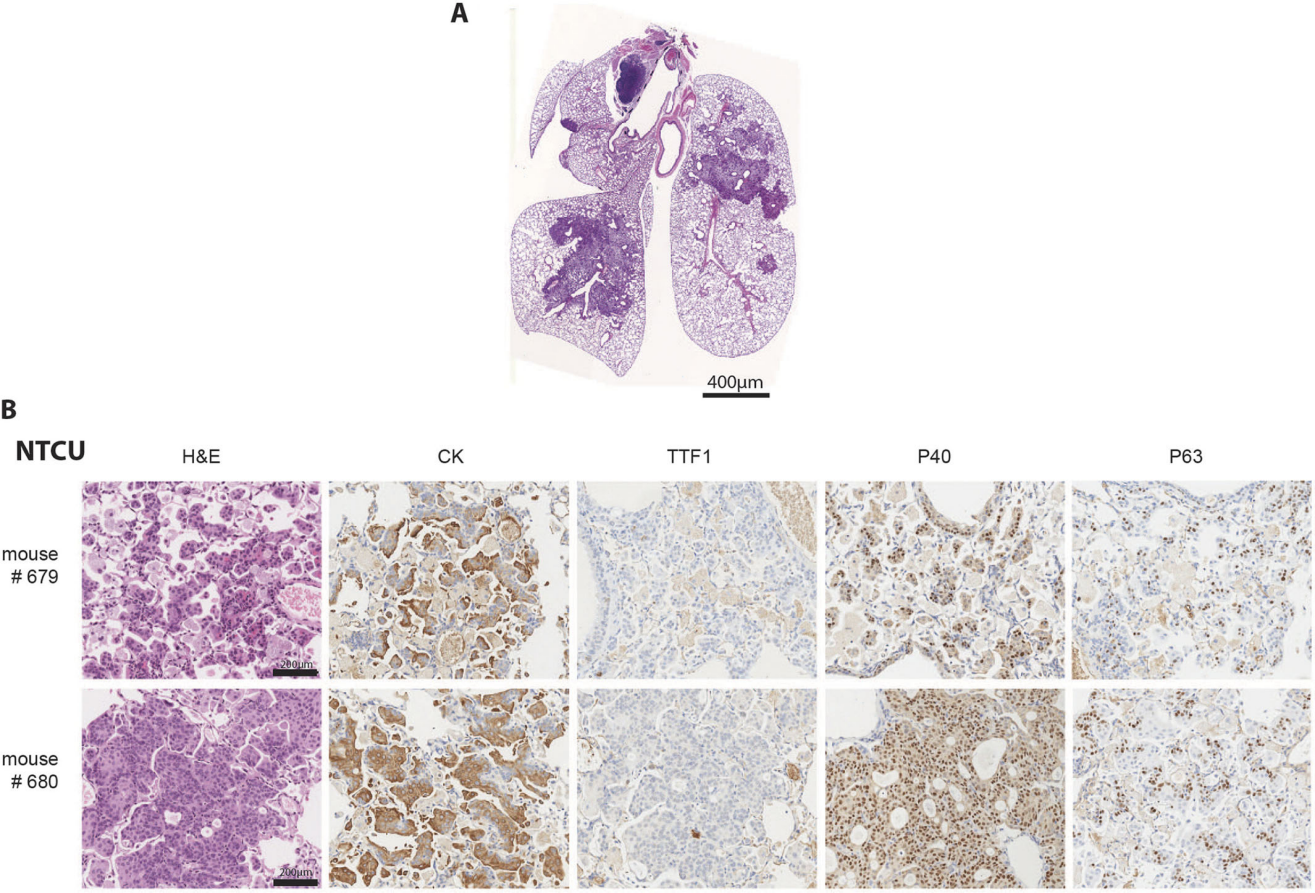
**Immunohistochemical characterization.** (A) Lungs of A/J mice bearing NTCU-induced tumors. Tumor location and morphology are consistent with squamous lung cancer (LUSC). Scale bar: 400 µm. (B) Histological sections of lung tumor lesions in the above mouse model. H&E staining shows typical LUSC morphology of tumor cells, and immunochemistry staining for cytokeratin (CK), thyroid transcription factor 1 (TTF1), P40 and P63 matches that of LUSC histology. Scale bars: 200 µm.

Exposure to the carcinogen NTCU is a known strategy to generate LUSC in mice (Wang et al., 2004). As observed for human LUSC carcinogenesis, preneoplastic lesions (dysplasia) and *in situ* carcinomas were observed in mouse bronchi upon NTCU treatment (Hudish et al., 2012). After 5 months of treatment with NTCU, A/J mice developed LUSC tumors, generally located in the central airways of the lung (Fig. 1A). Hematoxylin & eosin (H&E)-stained histological sections of lung tumors from these mice showed lesions with the typically differentiated stratified squamous epithelium. The immunohistochemical characterization of the NCTU-derived tumors showed their squamous carcinoma histotype. The lesions were positive for cytokeratins, showing epithelial origin, and for p40 and p63, hallmarks of LUSC tumors (Fig. 1B). Moreover, lesions did not stain with antibodies against the adenocarcinoma subtype marker TTF1. Two cell lines – UN-SCC679 and UN-SCC680 – were obtained out of 50 processed tumors from two different mice. Both cell lines were transplanted into the flank of immunosuppressed Rag^−/−^ IL2Rg^−/−^ mice, generating an allograft model. Subsequently, a syngeneic model was developed in A/J mice from which the two final working cell lines were established. Both UN-SCC679 and UN-SCC680 were fusiform, adherent cells with similar and fast *in vitro* proliferation rates (Fig. S1B and C). Progressive de-differentiation of the typical squamous phenotype was observed with increasing passages, as previously reported for human lung LUSC cancer cell lines (Hazawa et al., 2017; Chen et al., 2018a).

### Whole-exome and RNA sequencing

**Fig. 2.**
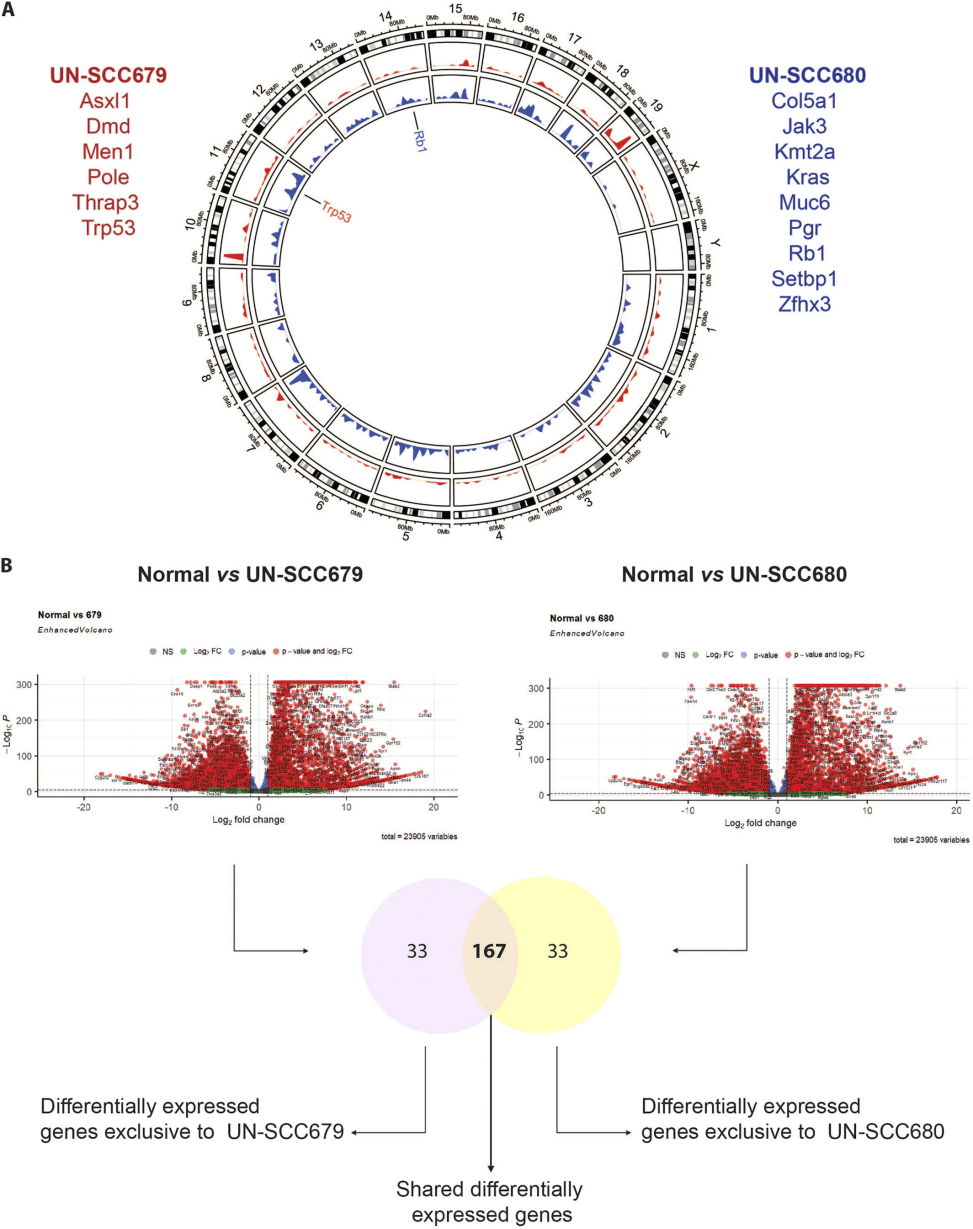
**Genomic characterization of UN-SCC679 and UN-SCC680 cell lines obtained from NTCU-induced lung tumors in A/J mice.** (A) Circle plot shows *Mus musculus* chromosome coordinates at the outside circular layer of the tumor. In the inside layers, mutation positions and frequencies in UN-SCC679 (red) and UN-SCC680 (blue) cell lines are plotted. Genes labeled in the circle plot represent those mutated in UN-SCC cell lines specific for LUSC. Listed in red are cancer-driving genes mutated in UN-SCC679. Listed in blue are cancer-driving genes mutated in UN-SCC680. (B) Flow chart comparing RNASeq data. First, data from each cell line (UN-SCC679 and UN-SCC680) were compared to those from normal lung. Differentially expressed genes are shown in a volcano plot, showing the significance versus the log_2_ fold change of gene expression on *y*- and *x*-axes, respectively. The *y*-axis represents the negative log of the *P*-value, the *x*-axis represents the log of the fold-change between the two conditions are shown. Red dots indicate genes with adjusted *P*-values <5×10^−5^ and log_2_ fold changes >2. Green dots indicate genes that do not meet the *P*-value requirement. Dots on the right side of the plots indicate genes that are overexpressed in the two UN-SCC cell lines when compared to control cells. Dots on the left side of the plots indicate genes that are downregulated in the two UN-SCC cell lines when compared to control cells. The Venn diagram shows genes that are differentially expressed in UN-SCC679 and UN-SCC680 cells. Common and different differentially expressed genes are listed in Table S5.

To characterize and compare the genomic alterations present in these two syngeneic LUSC cell lines, whole-exome sequencing was performed. The type and frequency of mutations for both cell lines are shown in Table 1. Being in the range of non-synonymous mutations consistent with human NSCLC data (Rizvi et al., 2015; Hammerman et al., 2012), UN-SCC679 cells showed a lesser mutational burden when compared to UN-SCC680 cells. Despite coming from the same mouse model and belonging to the same histological subtype, UN-SCC679 and UN-SCC680 cells presented different mutational profiles (Fig. 2A). In fact, they just shared alterations in seven widespread genes, i.e. *Aplnr*, *Fbn1*, *Dnah6*, *Cdh23*, *Trhde*, *Csmd3* and *Csf2rb*. UN-SCC679 cells showed a mutation in *Tp53* and UN-SCC680 cells in *Rb1*, both tumor suppressor genes characteristic of LUSC in patients (Bhateja et al., 2019). In addition, other oncogenes or tumor suppressor genes that are present in human tumors according to Bailey et al. (Bailey et al., 2018), were found (Fig. 2A). A complete list of mutated genes is shown in Tables S1 and S2.

**Table 1.**
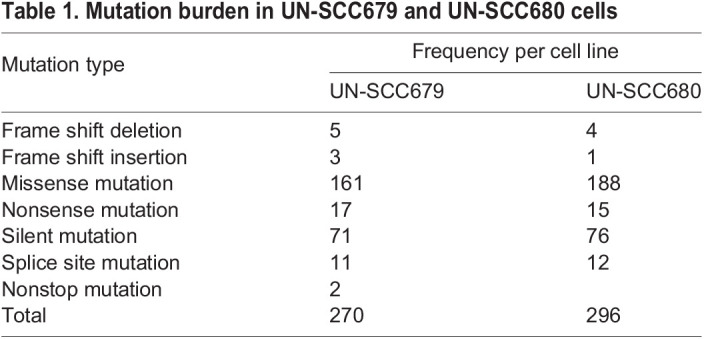
Mutation burden in UN-SCC679 and UN-SCC680 cells

### Comparative transcriptomics

We also studied the comparative gene expression profile of UN-SCC679 and UN-SCC680 cells by using RNA sequencing, using mouse lung non-malignant epithelial basal cells tissue as reference. Differentially expressed genes with an adjusted restrictive *P*-value <5×10^−5^ are represented in Fig. 2B, and listed in Tables S3 and S4 (Tables S3 and S4). We compared the differentially expressed genes identified in UN-SCC679 with those in UN-SCC680 cells. We observed that ∼80% of the differentially expressed genes are present in both cell lines. This indicated that, despite their differences in mutational patterns, UN-SCC679 and UN-SCC680 cells share many molecular similarities that reflect their identical squamous origin. Shared and non-shared differentially expressed genes are listed in Table S5. Compared to normal lung tissue, we found that both cell lines are enriched in p63 (compared to whole lung murine tissue; GSE118246) and that expression of genes related to the classic subtype described for human LUSC genomic subtypes, such as *Gsto1*, *Aldh3a1*, *Bcl6*, *Atp5g3*, *Dld*, *Odc1*, *Trp63*, *Gsta4*, *Ndufb5*, *Ephx1* or *Cox5b* (data not shown) is increased (Cancer Genome Atlas Research Network, 2012). Another characteristic of the classic subtype is the alteration of PI3K and KEAP1 pathways. We found that, compared to basal epithelial cells, *Keap1* and *Pik3ca* are enriched in both cell lines (Fig. S1E). Finally, both cell lines overexpress *Arnt2*, which has been previously associated to lung cancer (Yang et al., 2015).

### Immunotherapy response

**Fig. 3.**
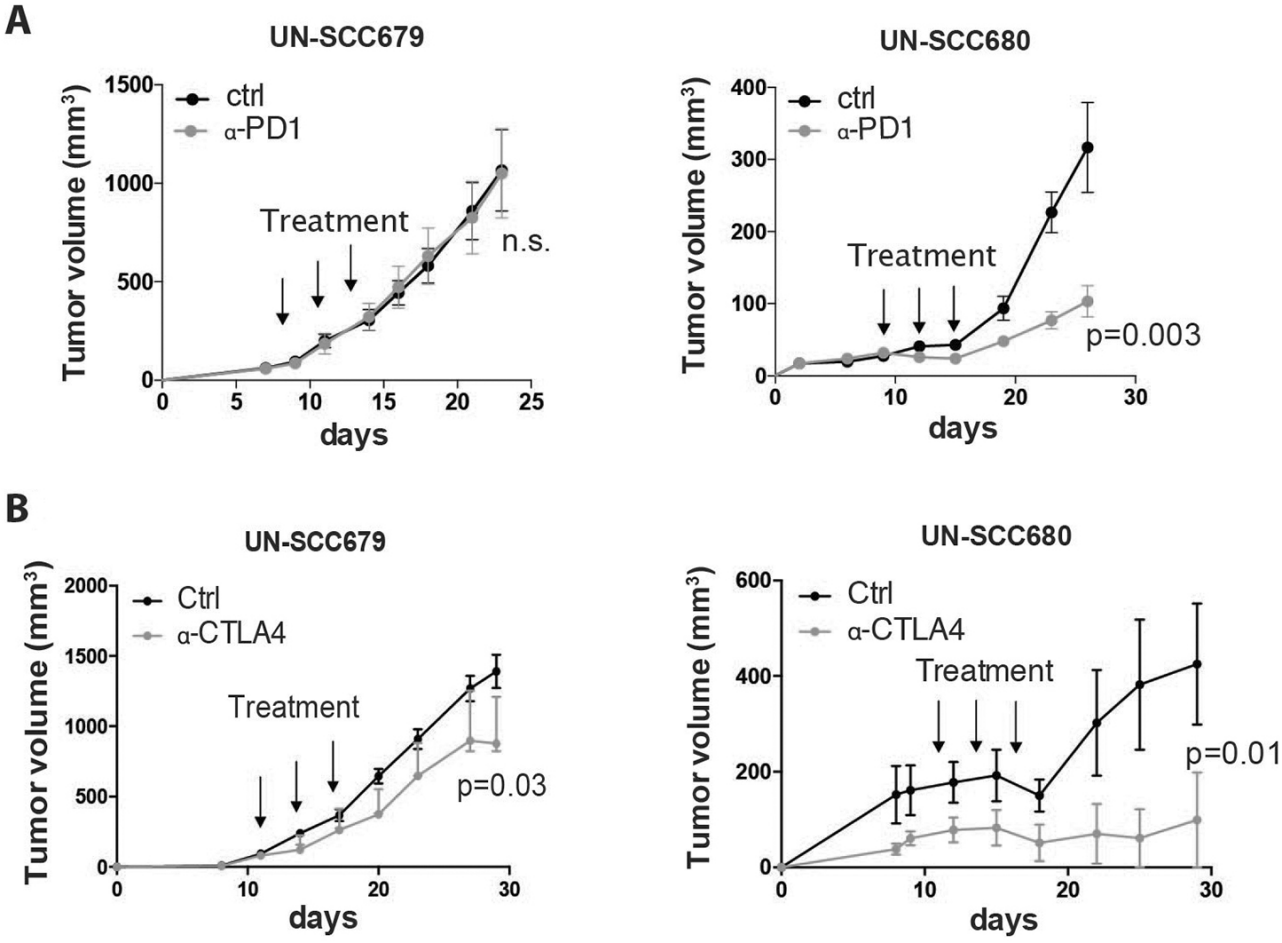
**Response to immunotherapy.** (A) Average tumor volume of isografts obtained from mouse UN-SCC679 and UN-SCC680 tumors treated with three doses of 100 µg of anti-PD-1 antibody (α-PD1) or PBS (ctrl) when they had reached a volume of 75 mm^3^ (*n*=6 per group). Significance was analyzed by *t*-test. (B) Average tumor volume of isografts from mouse UN-SCC679 and UN-SCC680 tumors treated with three doses of 100 µg of anti-CTLA4 (α-CTLA4) or PBS when they had reached a volume of 75 mm^3^ (*n*=6 per group). Significance was analyzed by *t*-test.

It has previously been reported that increased tumor mutation burden is associated with clinical efficacy of inhibition of programmed cell death 1 (PDCD1, also known as PD-1), i.e. anti-PD-1 therapy (Rizvi et al., 2015). Taking in consideration the mutational differences found between tumors of UN-SCC679 and UN-SCC680 cells, we wanted to assess and compare the effect of anti-PD-1 therapy and therapy against cytotoxic T lymphocyte-associated antigen (CTLA4), i.e. anti-CTLA4 therapy, in both LUSC models. Although proliferation rates of UN-SCC679 and UN-SCC680 cells was similar *in vitro* and *in vivo* in immune-deficient mice (Fig. S1C and D), UN-SCC679 cells showed an advantageous *in vivo* tumor growth in immune-competent mice compared to that of UN-SCC680 cells (Fig. 3A,B), suggesting that UN-SCC680 cells provoke an increased immune response compared with UN-SCC679 cells. In fact, 10-20% of mice inoculated with UN-SCC680 cells showed spontaneous regression of their tumors. Moreover, anti-PD-1 therapy significantly reduced UN-SCC680 tumor growth, whereas UN-SCC679 tumors showed complete resistance (Fig. 3A). These results are consistent with the lower mutational burden of UN-SCC679 cells compared to that of UN-SCC680 cells. However, although both UN-SCC679 and UN-SCC680 tumors tended to partially respond to anti-CTLA4 therapy (Fig. 3B), the response of UN-SCC680 tumors seemed more robust than that of UN-SCC679 tumors. Interestingly, the UN-SCC679 and UN-SCC680 models resemble the clinical reality of 15:85 patients who are responsive or resistant to immunotherapy.

### Immune landscape characterization

**Fig. 4.**
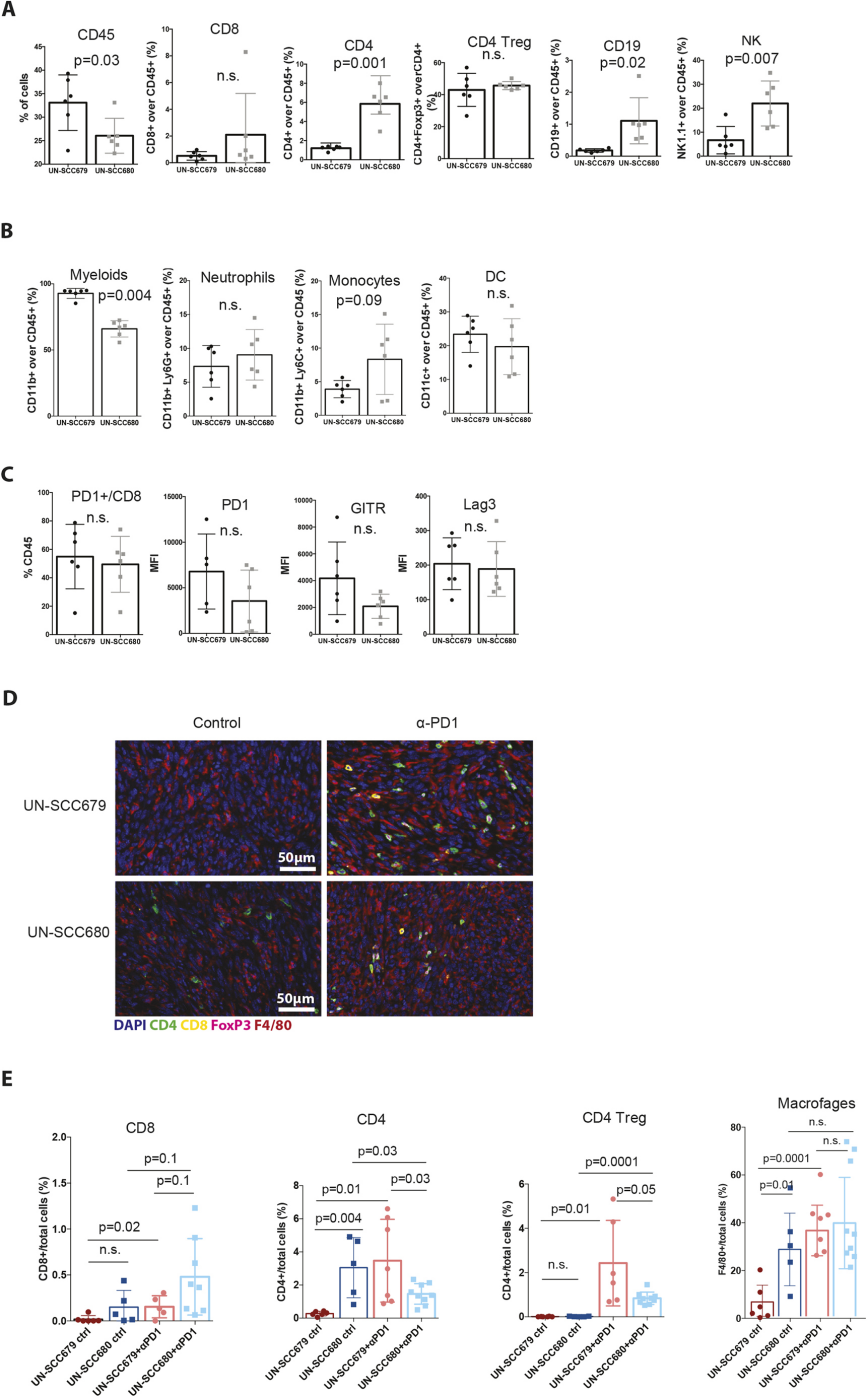
**Immune landscape characterization.** (A) Relative quantification of CD45^+^ and lymphoid cells infiltrating UN-SCC679 and UN-SCC680 tumors. (B) Relative quantification of myeloid cells infiltrating UN-SCC679 and UN-SCC680 tumors. (C) Relative quantification of immune exhaustion markers CD8+ cells infiltrating UN-SCC679 and UN-SCC680 tumors. Plotted is the Median Fluorescence Intensity (MFI). Analyses were made at day 13 post inoculation (*n*=6 per group). Significance was analyzed by *t*-test. (D) VECTRA images showing the main immune cell populations infiltrating UN-SCC679 and UN-SCC680 tumors that had been control treated or treated with anti-PD-1 antibody (α-PD1). (E) Relative quantification of CD8, CD4, CD4 Treg (defined as CD4+, FOXP3+) cells and macrophages (F4/80+) infiltrating UN-SCC679 and UN-SCC680 tumors that had been control treated or treated with anti-PD-1 antibody represented as target cells/total cells in percent. n.s., not significant.

The success of checkpoint blockade in NSCLC, both squamous cell carcinoma and adenocarcinoma, is strongly influenced by the nature of the immune infiltrate present in the tumor (Cristescu et al., 2018). To better understand and compare the types of immune response against our cell line models of squamous tumors, we analyzed the immune infiltrates of these LUSC models by flow cytometry. We chose day 13 post inoculation because, at that point, UN-SCC679 and UN-SCC680 tumors were similar in size and, thus, still comparable. UN-SCC679 tumors had a higher percentage of immune cell infiltration (Fig. 4A). This was dominated by myeloid cells (Fig. 4B) and, in particular, by tumor-associated macrophages (Fig. 4B). In contrast, UN-SCC680 tumors were infiltrated by significantly fewer myeloid cells and showed bigger proportions of anti-tumor immune cell populations [CD8T cells, non-regulatory T (non-Treg) CD4T cells and NK cells] infiltrating the tumor microenvironment. Although the percentage of PD-1 positive CD8T cells did not differ between the two tumor models, the mean fluorescence intensity of both PD-1 and GITR on CD8T cells was slightly lower in the CD8T cells infiltrating UN-SCC680 tumors, suggesting a less-exhausted phenotype in CD8T cells (Fig. 4C). Overall, these data indicate that, within our models, the two cell lines generated two different microenvironmental landscapes. In UN-SCC680-derived tumors the immune infiltrate is characterized by a stronger anti-tumor immune response, which is consistent with a greater response to treatment with anti-PD-1 antibody and a higher tumor mutational burden in this cell line. However, UN-SCC679 cells seem to induce a more-immunosuppressive environment.

We completed our immune population study by performing a multiplexed immunohistochemistry experiment using VECTRA technology (Fig. 4D and E). There, our FACs data were confirmed, as we observed the same trends in CD8, CD4, CD4 Treg and macrophages. In addition, we assessed immune populations changes after anti-PD-1 treatment. We observed an increase of CD8+ cells infiltration, more pronounced in UN-SCC680 cells, an increase in macrophages and, interestingly, a significant increase in CD4 Tregs in UN-SCC679 cells treated with anti-PD-1 antibody, a fact that might relate to decreased response to checkpoint inhibitors and poor disease prognosis (Saleh and Elkord, 2019; Cai et al., 2019; Kamada et al., 2019).

### Metastatic ability and organotropism

**Fig. 5.**
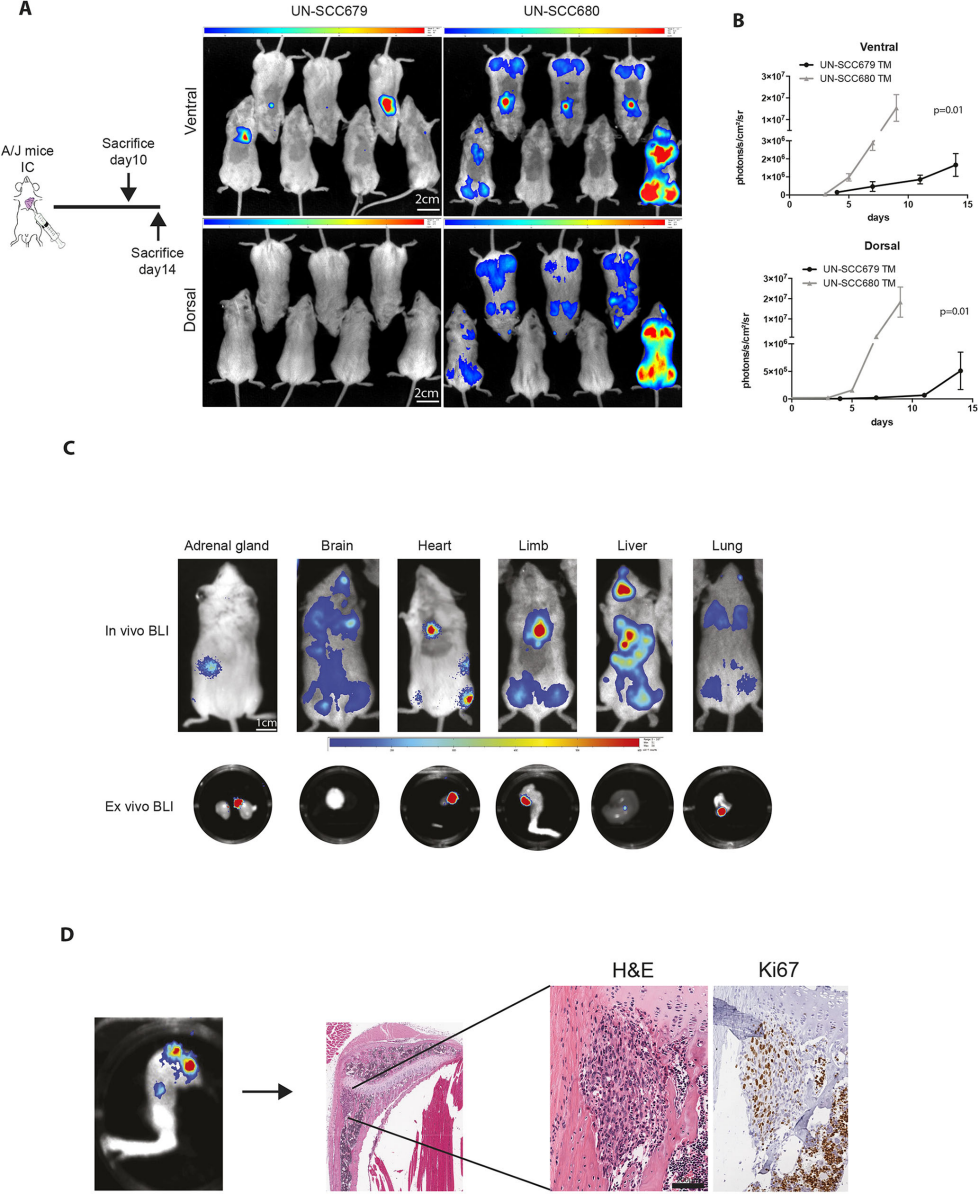
**Metastatic features of UN-SCC679 and UN-SCC680 cell lines derived from NTCU-induced tumors.** (A) Left: Schematic outline of the experiment. Signs of morbidity (cachexia or reduced mobility) were used as the endpoint for the experiment. Right: Representative bioluminescence images of mice in ventral and dorsal position at day 7 after intracardiac inoculation. Scale bars: 2 cm. Color bars: ventral min 6.41, max 45 counts; dorsal min 3.39, max 17.4 counts. (B) Quantification bioluminescence imaging (*n*=7 per group was analyzed by *t*-test). (C) Representative bioluminescence images (BLI) showing tumor cells in different metastatic organs. Top: Whole *in vivo* mice images. Bottom: Luminescence in *ex vivo* organs. Scale bar: 1 cm. Color bars: min 0.1x10^−3^, max 0.6x10^−3^ counts. (D) Representative images of a hindlimb. Tumor burden in histological sections from an UN-SCC680-bearing mouse was assessed by staining with H&E and Ki-67. Scale bar: 200 µm.

Next, we assessed the metastatic ability of UN-SCC679 and UN-SCC680 cells. In the NTCU-induced model, we observed the appearance of metastases in axillary nodes, bone, liver and heart (data not shown). To explore the specific metastatic potential of the cell line models, we inoculated the left heart ventricle of A/J mice with 1×10^5^ UN-SCC679 or UN-SCC680 cells that had been transfected with a triple modality GFP-luciferase-thymidine reporter gene (Fig. 5A). Thus, our experimental design recapitulated the metastatic events that occurred after cells were shed from the primary site into the systemic circulation (extravasation, homing and colonization). Bioluminescence images showed tumor cells in lungs, limbs, adrenal glands, liver and brain. On average, UN-SCC680 cells engrafted a week earlier than UN-SCC679 cells, which suggest a more-aggressive phenotype and, thus, a difference in the metastatic potential between the two elements of our cell line models. In fact, UN-SCC680-inoculated mice showed signs of morbidity at day 10, whereas UN-SCC679-inoculated mice did not show morbidity signs until day 14 post inoculation. Quantification of mouse bioluminescence showed the same trend in ventral and dorsal position, and was significantly higher in the UN-SCC680 than the UN-SCC679 model (Fig. 5B). At the experimental end point, we extracted different organs and verified luminescence of tumor cells *ex vivo* (Fig. 5C). We found tumor cells in hindlimbs, heart, lungs, liver, adrenal glands and brain. Histological sections were obtained from hindlimbs to demonstrate the presence of a metastatic lesion (Fig. 5D). These data demonstrate a metastatic organ pattern in both cell lines, which seems seemingly identical to that in human LUSC.

## DISCUSSION

To obtain a preclinical model of LUSC used to be a tedious and long process of chemical induction by carcinogens, i.e. the intratracheal instillation of chemicals (Whitmire et al., 1981; Yoshimoto et al., 1977), or topical application of N-nitroso-methyl-bis-chloromethyl urea or N-Nitroso-tris-chloroethylurea (NTCU) (Wang et al., 2004). More recently, the generation of different GEMMs, accomplished by targeting single or combined driver mutations to distinct cells of origin, provided less-cumbersome tools for cancer researchers (Ferone et al., 2020). However, chemically induced lung cancer models present several advantages over GEMMs, as the spectrum of mutations achieved in these models is wider and more heterogeneous, thereby mimicking human pathologies. This is especially true in tobacco-associated subtypes, such as LUSC.

The number of cell lines derived from LUSC animal models is still very limited (Kaneko and LePage TKS, 1980). Besides, thorough molecular and functional characterization of these cell lines is crucial, so they can be used by the scientific community with a broad knowledge of their more-relevant traits. This is key for any cell model that aims to be a quality tool providing relevant results. Lung cancer research has been one of the pioneer fields in valuing this characterization for cell line models (Mulshine et al., 2020). Functional aspects, such as level of proliferation, immunogenicity, metastatic capacity and organotropism, are particularly relevant when selecting a cancer cell line model.

Despite coming from the same murine carcinogenesis model, having been generated in the same experimental setting and sharing the same immunohistochemical traits for LUSC cells, the two cell lines derived from the tumors that comprise our two cell lines models – namely UN-SCC679 and UN-SCC680 cells – present different genotypic and phenotypic characteristics. This offers the opportunity to use two alternative models for different comparative analytical purposes.

Amongst the relevant differences we found in these two cell line models, the disparity of mutated genes as well as the different gene expression profiles were relatively unexpected. Still, although the number of mutated genes that are shared is not high, i.e. only present in seven genes, all mutated genes are relevant in human lung cancer. For example, expression of the G protein-coupled receptor family member apelin receptor (*APLNR*) has been shown to promote proliferation and cell autophagy via ERK1/2 signaling in human lung cancer cells (Yang et al., 2014). Dynein axonemal heavy chain 6 (*DNAH6*) belongs to the dynein family, whose members encode large proteins that constitute the microtubule-associated motor protein complex; it has recently been related to smoking-associated lung cancer (Chen et al., 2018b). Besides, analysis of lung cancer patient data indicated that cadherin related 23 (*CDH23*) – a member of the cadherin superfamily, whose genes encode calcium-dependent cell-cell adhesion glycoproteins – is downregulated in lung cancer, working as a suppressor of cell migration (Sannigrahi et al., 2019). Several groups have proposed CUB and sushi multiple domains 3 (*CSMD3*) to be a frequently mutated gene in human LUSC, resulting in an increased proliferation of airway epithelial cells (Cancer Genome Atlas Research Network, 2012; Liu et al., 2012; Li et al., 2015). Fibrillin-1 (*FBN1*), encoding an extracellular matrix glycoprotein that serves as a structural component of calcium-binding microfibrils, seems to play an important role in tumor-related immune infiltration and has been proposed as a prognostic and predictive biomarker for immune therapy against pancreatic ductal adenocarcinoma (PDAC) (Luan et al., 2020). Finally, colony stimulating factor 2 receptor subunit beta (CSF2RB) has been shown to regulate epithelial-mesenchymal transition (EMT) in human lung cancer cells (Wu et al., 2020; Rudisch et al., 2015). Our study also shows mutations in genes that are involved in mechanisms relevant in cell-cell adhesion, microtubule activity or extracellular matrix conformation, thereby indicating the presence of commonly altered functions in LUSC tumorigenesis.

Relevant LUSC studies coincide in emphasizing the molecular heterogeneity of this tumor type, which makes the development of targeted therapies very difficult (Hammerman et al., 2012). Thus, we analyzed to which of the described genetic human LUSC subtypes our two mice LUSC cell lines could be assimilated, according to their gene expression profiles. Comparing our RNASeq data to what has been published about human LUSC genomic subtypes (Cancer Genome Atlas Research Network, 2012), both cell line models are enriched in the expression of genes related to the human ‘classic’ LUSC subtype.

The National Comprehensive Cancer Network guidelines for LUSC treatment (Ettinger et al., 2019) do not recommend any specific molecular testing for LUSC. This seems to be a consequence of the above-mentioned lack of common genetic alterations in LUSC and the lack of actionable mutations. However, immunotherapy has been an emerging and promising treatment for LUSC patients. The approval of the anti-PD-1 antibody pembrolizumab as a first-line treatment in selected patients has made PD-L1 immunohistochemistry mandatory for all patients with advanced NSCLC that lacks sensitizing mutations regarding targeted therapies.

It is well stablished how specific mutations drive immune evasion in cancer. Specifically, mutations in *KRAS* and *P53* are partly responsible for the resistance of lung tumors to immunotherapy (Spranger and Gajewski, 2018; Ischenko et al., 2021). UN-SCC679 cells showed mutated *p53* (the most common alteration in human LUSC), which could be involved in its resistance to anti-PD-1 treatment. However, UN-SCC680 cells, which responded to immunotherapy to some extent, contained mutated *Kras*. Nevertheless, the specific *Kras* mutation found in UN-SCC680 cells was a rare alteration of the gene, which differed to the classic G12V/D mutation that characterizes lung cancer. This detail must be taken into account, since *Kras* is not a gene characteristically mutated in LUSC and because the mutation we found in our cell line might have other functional and biological implications regarding the tumoral context, which are different to those expected with the classic G12V/D mutation.

In the context of sensitivity to immunotherapy and based on the tumor immune landscape, six immune subtypes (ISs) have been identified in SCC. The most common for LUSC – comprising ∼41% of cases – is IS1, followed by IS5 (25%) and IS3 (14%). Both IS1 and IS3 are ‘immune-cold’, characterized by a low lymphocyte infiltration rate, a high immunosuppressive microenvironment and a poor prognosis. IS3 has the worst outcome among all subtypes (Li et al., 2019). According to this classification, the immune infiltrate we describe in our present study for UN-SCC679 tumors could be assimilated to the features of the IS3 subtype, with a rich immunosuppressive microenvironment characterized by myeloid-derived immune suppressor cells. However, UN-SCC680 tumors were infiltrated by significantly fewer myeloid cells and had bigger anti-tumor immune cell populations, comparable to IS1. Thus, our cell line models can be used to experimentally compare two different immune landscapes found in human LUSC.

The immune landscape and mutational burden have been shown to predict the response of LUSC to immunotherapy. In general, a tumor with a high lymphocytic infiltrate and an elevated mutational burden will have a greater response to immunotherapy treatments (Rizvi et al., 2015). UN-SCC679 and UN-SCC680 cell lines generate tumors with different immune profiles that respond differently to classical immunotherapies, UN-SCC680 being significantly more sensitive than UN-SCC679. Thus, this pair of cell lines represents a highly valuable system to study new immunotherapy agents or new combined treatments.

A well-known feature of human lung tumors is their selective metastatic tropism. Lung cancer most commonly spreads through bloodstream to the liver (34.3%) and adrenal glands (32.6%), followed by bones (14.9%) and other organs, such as the central nervous system (CNS) (12%), kidney (10.9%), myocardium (9.1%), pancreas (5.1%), spleen (4%), small or large intestines (3.4%), stomach (2.3%), thyroid gland (1.7%) and ovary (0.6%) (Milovanovic et al., 2017). The two cell lines described here showed a metastatic potential that is new in the field. In our models, we observed a clear tropism for bone (mainly high limbs), brain and, less frequently, liver and adrenal glands. We are currently developing metastatic cell lines derived from UN-SCC679 and UN-SCC680 cells with enriched tropism for the above-mentioned organs. Finally, UN-SCC680 cells showed a more-aggressive phenotype compared to that of UN-SCC679 cells, which is consistent with greater immunogenicity and increased mutational burden.

In conclusion, we have generated and thoroughly characterized two mouse LUSC cell models, i.e. cell lines UN-SCC679 and UN-SCC680 derived from the NTCU-induced mouse model in A/J mice. UN-SCC679 and UN-SCC680 cells carried different somatic mutations and gene expression profiles, with tumor mutation burdens comparable to those of LUSC patients. UN-SCC680 cells responded partially to treatment with anti-PD-1 antibody, whereas UN-SCC679 cells were refractory to the treatment. The immune landscape study revealed a cold IS of both cell lines, with a more-immunosuppressive tumor microenvironment in UN-SCC679 tumors. Finally, both cell lines demonstrated metastatic potential with the ability to grow metastatic lesions in the brain, bones, liver and adrenal glands of different degree of aggressiveness. In summary, we believe we have generated a very valuable tool for further LUSC research, comprising two cell lines with complementary traits that recapitulate the complexity of LUSC in humans.

## MATERIALS AND METHODS

### Mice

Seven-week-old A/JOlaHsd mice (hereafter referred to as A/J mice) were obtained from Harlan Laboratories (Harlan-Winkelmann). All animal experiments were conducted in accordance with the standards set by the Declaration of Helsinki. The protocols were approved by the institutional animal care committee of our institution (approvals 049-18 and 035c-20), following the legal and ethical requirements demanded by the European Communities Council Directive 2010/63/UE and according to the regulations of the Spanish Royal Decree 53/2003, which were drawn up to ensure that pain and suffering would be limited to the lowest level.

### Carcinogenesis and cell line generation

LUSC tumors were induced by N-nitroso-tris-chloroethylurea (NTCU; Toronto Research Chemicals) treatment applying 0.04M NTCU by skin painting twice a week for 20 weeks to 8-week-old A/J mice. Lungs of euthanized mice were excised, and tumor cell lines UN-SCC679 and UN-SCC680 were derived and cultured for 25 passes to ensure they were immortalized. Cells were then subcutaneously injected into the flanks of 6-week-old female Rag2^−/−^IL2Rg^−/−^ immunodeficient mice and engrafted into syngeneic 8-week-old A/J mice as previously described (Azpilikueta et al., 2016) (Fig. S1A). Both cell lines were cultured in RPMI 1640 supplemented with 10% Fetalclone serum (Thermo Fisher Scientific) and 100 U/ml penicillin-100 µg/ml streptomycin (Thermo Fisher Scientific). All cells were grown in a humidified incubator under 5% CO_2_ at 37°C. Cell lines were routinely tested for mycoplasma.

### Immunohistochemical staining

Dissected tumors were fixed in 4% paraformaldehyde and embedded in paraffin. Three-micrometer-thick sections were used for tumor immunohistochemical characterization. Briefly, sections were deparaffinized, hydrated through a graded series of ethanol, and endogenous peroxidase activity was quenched with 3% hydrogen peroxide. Antigen retrieval and primary antibody conditions were as follows: CK cocktail (BioGenex), catalog no.: AM071-10M, 1:50, TE pH 9.0, 20 min at 95°C; TTF1 (Dako) catalog no.: M357529-2, 1:40, TE pH 9.0, 6 min at 98°C; P40 (Biocare Medical) catalog no.: 3066, 1:200, TE pH 9.0, 20 min at 95°C; P63 (Abcam) cataloge no.: ab735, 1:100, EDTA pH 8.0, 20 min at 95°C. All primary antibody incubations were carried out overnight at 4°C. After detection of primary antibodies was performed with EnVision System (Dako), slides were counterstained with hematoxylin, dehydrated, and covered with DPX mounting medium.

### Exome sequencing

Whole-exome sequencing was performed on extracted DNA from the tumor-derived cell lines. Enrichment for sequencing was performed using the SureSelectXT Mouse All Exon kit (Agilent) following the manufacturer's protocol. Exon-enriched libraries were subjected to paired-end sequencing, 2×100 base pairs read length and 60× minimum coverage.

Raw sequencing reads were aligned to the respective mouse reference genome (NCBI37/mm9). Alignment was performed with the BWA aligner. Concordant read-pairs were identified as potential PCR duplicates and subsequently masked in the alignment file. Somatic mutations and copy number alterations were determined using our in-house analysis pipeline (Peifer et al., 2012; Fernandez-Cuesta et al., 2014). Data are available on the University of Cologne Scientific Dataset public repository website at https://uni-koeln.sciebo.de/s/JJdOLcanGNN7U92.

### RNA sequencing analysis

Samples were prepared with the Illumina TruSeq Stranded mRNA kit following the manufacturer's instructions and sequenced as reverse paired-end (50 bp) runs on the Nextseq sequencer. For RNA sequencing analysis, raw fastq files were trimmed with Trimmomatic/0.36. Pseudoalignment was carried out to the mm10 reference genome and gene level counts were determined with Kallisto (Bray et al., 2016). Differential gene expression analysis was conducted with R/4.0.3. Samples were imported, normalized and analyzed with the DESeq2 package. To compare UN-SCC cell to normal lung basal cells, transcriptomic information from GSE83991, entitled “Transcriptome analysis of mouse lung epithelial cells”, was used. Data are available on GEO website under accession number GSE185054.

### Tumor growth measurements using *in vitro* cell proliferation assay

Cell proliferation in 2D was assessed using the CellTiter 96 AQueous Non-Radioactive Cell Proliferation Assay, MTS (Promega). Experiments were carried out on the days indicated, according to the manufacturer's instructions and normalized to day 1 post seeding. For *in vivo* tumor growth studies, 2×10^6^ UN-SCC679 or UN-SCC680 cells were subcutaneously inoculated in both flanks of 8-week-old female Rag^−/−^ IL2Rg^−/−^ mice (Harlan-Winkelmann). Tumor volume (weight^2^×length×0.52) was measured with a digital caliper twice a week until sacrifice.

### Tumor immunotherapy

2×10^6^ UN-SCC679 or UN-SCC680 cells were subcutaneously inoculated in one flank of 8-week-old female A/J mice (Harlan-Winkelmann). When tumors reached a volume of 75 mm^3^, mice were randomized (*n*=6 per group) and treated intraperitoneally with three doses of 100 µg of the following antibodies: anti-PD-1 (RMP1-14, BioXCell), anti-CTLA4 (9D9, BioXCell) or PBS every 3 days from day 7 post inoculation. Tumor volume (weight^2^×length×0.52) was measured with a digital caliper twice a week until euthanasia.

### Tumor immune landscape analysis

2×10^6^ UN-SCC679 or UN-SCC680 cells were subcutaneously inoculated in one flank of 6-8-week-old female A/J mice (Harlan-Winkelmann). Thirteen days after cell inoculation, tumors were collected and processed for flow cytometry analysis as previously described (Azpilikueta et al., 2016). Single-cell suspensions were treated with Fc block (2.4G2; BD Pharmingen) to avoid unspecific staining and then stained with the following fluorochrome-conjugated antibodies, as previously reported (Ajona et al., 2020). Data acquired in a FACSCanto II flow cytometer were analyzed using FlowJo software (Tree Star, Inc., Ashland, Oregon). The gating strategy used to analyze the data is shown in Fig. S2.

### Multiplexed immunohistochemistry

Formalin-fixed paraffin-embedded tissues from UN-SCC679 and UN-SCC680 subcutaneous tumors were used for multispectral immunodetection of CD4, CD8, FOXP3, F4/80 and DAPI (for nuclear staining) by using the murine-specific kit (NEL840001KT) from Akoya Biosciences (Marlborough, MA, USA) following the manufacturer's instructions. Extra Alexa Fluor tyramides Opal 540 (FP1494001KT), 620 (FP1495001KT) and 650 (FP1496001KT) from Akoya Biosciences were used as well. Citrate buffer pH6 was used for all antigen retrievals and removal of primary-secondary complexes formed with previous antibodies. Primary antibodies and its corresponding Opal reagents were as follows: FOXP3 (CST, 12653) at 1:600 dilution and combined with Opal 540 fluorophore; CD4 (Abcam ab183685), 1:400 dilution, Opal 520; CD8 (CST, 98941), 1:500 dilution, Opal 620; and F4/80 (CST, 70076), 1:400 dilution, Opal 650. Acquisition of multispectral images and unmixing were conducted with the Vectra Polaris Automated Quantitative Pathology Imaging System, using the Phenochart and InForm softwares (all Akoya Biosciences).

Multispectral analysis was performed using QuPath 0.3.0 software (Bankhead et al., 2017). Tumor regions were segmented manually. Then, cell limits were determined based on the DAPI staining (1.5% threshold and 5 μm nucleus expansion) and indexed as independent objects. QuPath object classifier algorithm was trained for the detection of the immune landscape and finally applied to the entire set of multispectral images. Density percentages were calculated using the number of positive cells for a specific immune population and divided by the total number of cells in the tumor.

### Mouse metastasis *in vivo* model

Cells had previously been transfected with a triple modality construct expressing GFP, luciferase and thymidine kinase (Ponomarev et al., 2004). Eight-week-old female mice (7 mice/group) were inoculated in the left cardiac ventricle with 1×10^5^ cells as detailed by Valencia et al. (2013). For bioluminescence (BLI), mice were anesthetized with a solution of ketamine (Imalgene, Merial) and xylazine (Rompun, Bayer AG), and intraorbitally injected in the eye with 50 μl D-luciferin 150 mg/ml (30 mg/kg bodyweight dissolved in PBS; Promega Benelux). Images were acquired immediately using a real-time *in vivo* system (PhotonImager, Biospace laboratory). For imaging analysis, M3 Vision software (v 1.1) was used. Photon flux was calculated for each mouse by using a circular region of interest for the whole body and extracted as photons/s/cm^2^/sr. All *in vivo* experiments were normalized to the luciferase signal at day 0.

### Statistics

Sample size was chosen based on similar experiments previously published by the authors. For comparison of two groups, sample normality and variance were explored (Shapiro–Wilk test and Levene test, respectively). Groups with normal distribution followed a two-tailed *t*-test. Non-normal samples were analyzed using the Mann–Whitney test (equal variances) or the Median test (unequal variances).

## Supplementary Material

Supplementary information
