# Supplementary material for: Two cell line models to study multiorganic metastasis and immunotherapy in lung squamous cell carcinoma
Source: Dis Model Mech. 2022 Jan 31;15(1):dmm049137. doi: 10.1242/dmm.049137 (PMC8822220; doi:10.1242/dmm.049137)
Supplement: Supplementary information [file dmm-15-049137-s1.pdf]

NTCU model: cell lines generation

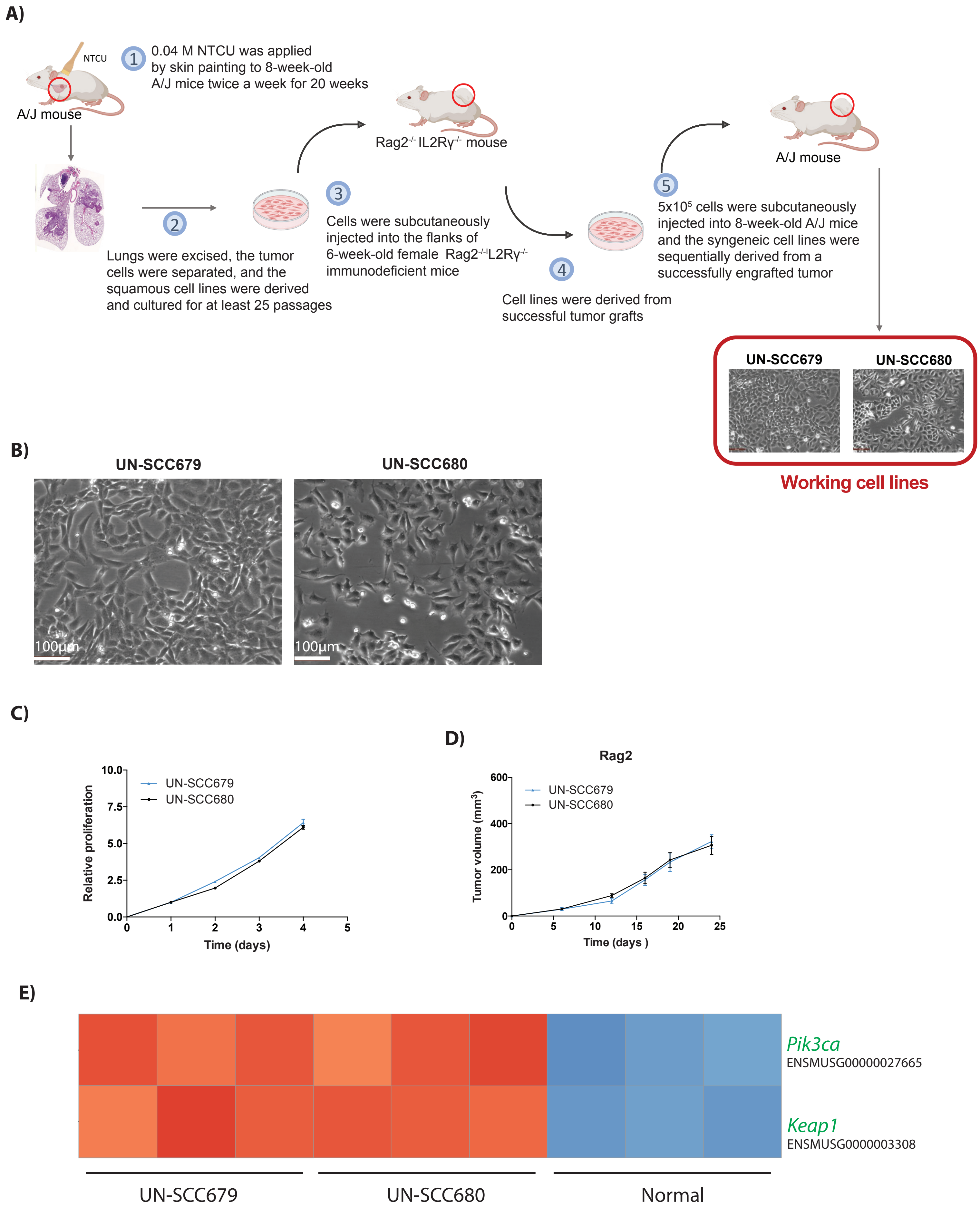

Fig. S1. UN-SCC679 and UN-SCC680 generation and proliferation rates.

A) Workflow of UN-SCC679 and UN-SCC680 cell lines generation from a NTCU mouse model.

B) 10x light-field images of cell lines grown in 2D showing its morphology.

C) MTS proliferation assay of UN-SCC679 and UN-SCC680 *in vitro* showed no growth differences between both cell lines.

D) *In vivo* proliferation assay using immunodeficient mice ($Rag2^{-/-}IL2Rg^{-/-}$) showed similar growth rates for UN-SCC679 and UN-SCC680.

E) Heap map showing *Keap1* and *Pik3ca* enrichment in both cell lines comparing to epithelial basal cells.

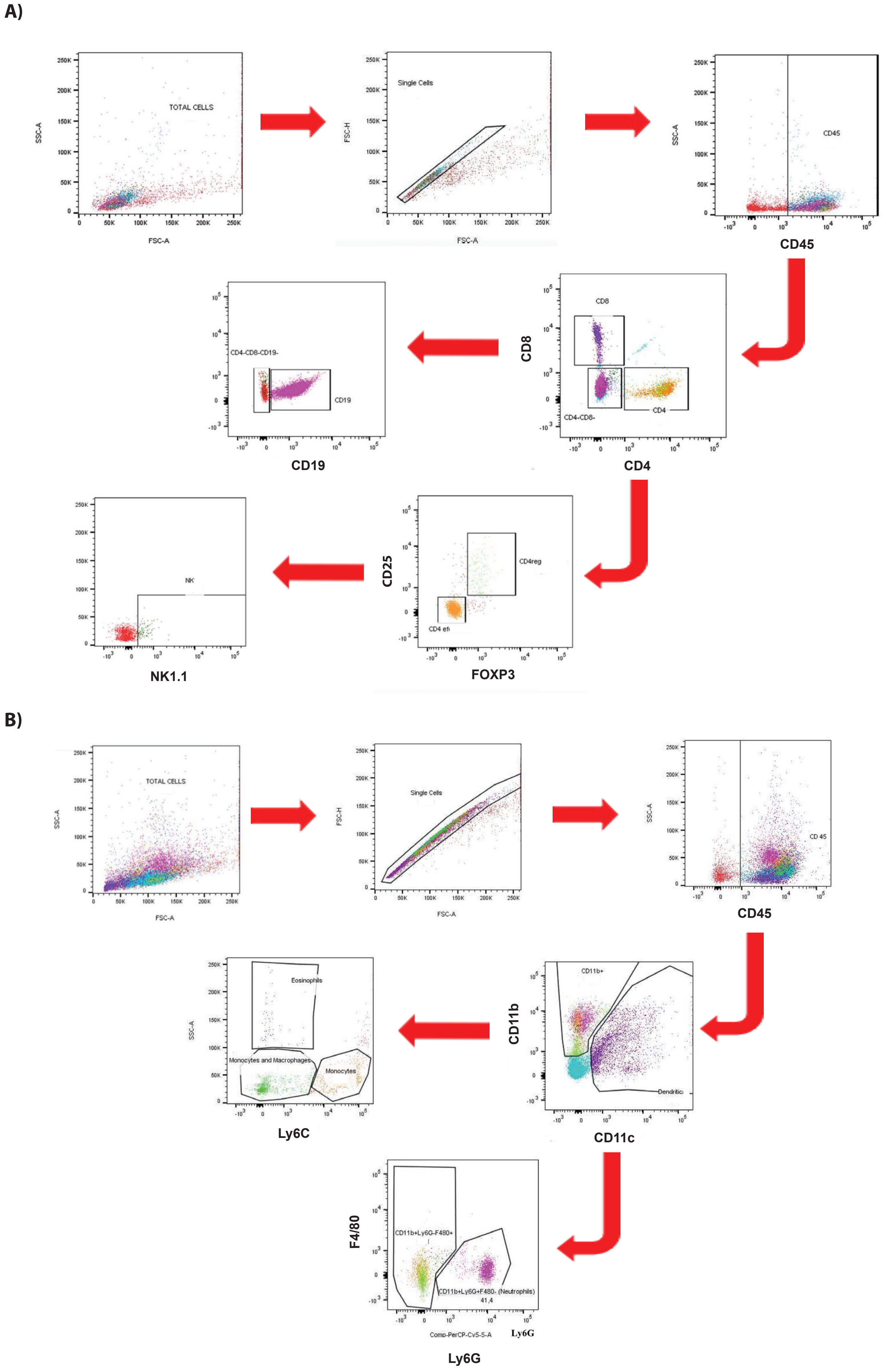

Disease Models & Mechanisms • Supplementary information

Fig. S2. Flow cytometry gating strategy for tumor infiltrating cells.
A) Lymphoid populations were separated from total tumor cells using a CD45 gate. CD8, CD4, CD19 and NK cells were separated from CD45-positive cells based on their corresponding marker expression.
B) For the identification of myeloid cells, CD11b-positive cells were gated and the proportion of Ly6C was evaluated. Neutrophils were considered Ly6G+, Ly6Clow; monocytes Ly6Chigh, F4/80low; tumor-associated macrophages (TAM) Ly6Clow, F4/80high and dendritic cells (DC)
CD11b-, CD11c+. Dead cells were not excluded.

Disease Models & Mechanisms • Supplementary information

Table S1. Mutated coding genes in UN-SCC679 cells

Name	Chr	Transcript	Mutation type	DNA change	Protein change	Allelic fraction
Aatk	11	NM_001198785	missense	c.790C>G	p.L264V	0.162162
Abca13	11	NM_178259	missense	c.14681G>T	p.C4894F	1
Abca9	11	NM_147220	silent	c.3429A>C	p.L1143L	0.111111
Acan	7	NM_007424	missense	c.5903T>G	p.F1968C	0.4
Actl9	17	NM_183282	missense	c.1213G>T	p.G405W	0.233333
Adipoq	16	NM_009605	missense	c.506T>G	p.V169G	0.336283
AF251705	11	NM_134158	silent	c.618C>G	p.V206V	0.104167
Ahnak	19	NM_009643	silent	c.660A>G	p.T220T	0.173653
Akip1	7	NM_020616	silent	c.189G>A	p.S63S	0.333333
Ankrd26	6	NM_001081112	missense	c.2341T>G	p.S781A	0.333333
Aoah	13	NM_012054	silent	c.918T>C	p.F306F	0.545455
Ap2b1	11	NM_001035854	missense	c.2305A>G	p.I769V	0.131818
Aplnr	2	NM_011784	silent	c.513G>A	p.L171L	0.696203
Appl2	10	NM_145220	missense	c.972C>A	p.D324E	0.606061
Appl2	10	NM_145220	missense	c.970G>A	p.D324N	0.59375
Armc5	7	NM_146205	silent	c.858T>A	p.A286A	0.285714
Arvcf	16	NM_033474	silent	c.2703C>T	p.Y901Y	0.378378
Asic3	5	NM_183000	silent	c.144A>G	p.T48T	0.592593
Asxl1	2	NM_001039939	missense	c.1460T>G	p.I487S	0.283333
BC048507	13	NM_001001185	silent	c.402C>A	p.V134V	0.337079
Bcar1	8	NM_009954	splice	c.924_splice	e5+2	0.785714
Bora	14	NM_175265	missense	c.433C>T	p.L145F	1
Cacna1g	11	NM_009783	silent	c.3483C>T	p.H1161H	1
Cadps	14	NM_012061	missense	c.2909A>C	p.Y970S	0.674074
Calcr	6	NM_007588	nonsense	c.1176C>G	p.Y392*	0.481481
Camkk2	5	NM_001199676	silent	c.441G>A	p.E147E	0.3
Capn10	1	NM_011796	missense	c.218G>T	p.C73F	0.444444
Cbfa2t2	2	NM_172860	missense	c.1742C>T	p.S581L	0.619469
Ccdc117	11	NM_134033	nonsense	c.253C>T	p.R85*	1
Ccdc152	15	NM_001166063	splice	c.430_splice	e6+2	0.168421
Ccdc7	8	NM_029061	splice	c.499_splice	e7-1	0.5
Ccdc96	5	NM_025725	missense	c.1823C>G	p.A608G	0.255556
Cdc14a	3	NM_001080818	missense	c.1160T>C	p.M387T	0.5
Cdh23	10	NM_001252635	missense	c.5629G>A	p.D1877N	0.5

Cdh3	8	NM_001037809	missense	c.854A>G	p.E285G	0.282353
Celsr3	9	NM_080437	silent	c.6621G>A	p.Q2207Q	0.62069
Chd6	2	NM_173368	missense	c.7700C>G	p.T2567R	0.317073
Cnga4	7	NM_001033317	missense	c.743T>C	p.V248A	0.321429
Cnr1	4	NM_007726	missense	c.2680C>T	p.R894C	0.578947
Cntnap3	13	NM_001081129	nonsense	c.2293G>T	p.E765*	0.37931
Cobl	11	NM_172496	silent	c.1251A>C	p.P417P	0.125
Col27a1	4	NM_025685	silent	c.786T>C	p.H262H	0.333333
Col6a3	1	NM_001243008	silent	c.7185A>C	p.V2395V	0.319588
Crybb2	5	NM_007773	silent	c.591G>A	p.Q197Q	0.681818
Csf2rb	15	NM_007780	missense	c.2096G>T	p.G699V	0.238095
Csmd1	8	NM_053171	missense	c.9100A>G	p.I3034V	0.179487
Csmd1	8	NM_053171	nonsense	c.9096C>A	p.C3032*	0.184211
Csmd3	15	NM_001081391	missense	c.10717G>A	p.E3573K	0.407407
Cspp1	1	NM_026493	silent	c.2673C>T	p.L891L	0.691489
Cxxc5	18	NM_133687	silent	c.444G>A	p.E148E	1
Cyp2c55	19	NM_028089	missense	c.881T>C	p.I294T	0.289855
Cyp3a25	5	NM_019792	frame_shift_del	c.324delT	p.F108fs	0.128472
Cypt12	3	NM_029289	missense	c.287A>G	p.E96G	0.21519
Dchs1	7	NM_001162943	silent	c.1062G>A	p.P354P	0.58427
Ddx54	5	NM_028041	missense	c.856G>A	p.E286K	0.6
Dgat1	15	NM_010046	missense	c.920T>G	p.I307S	0.25463
Dhrs13	11	NM_183286	missense	c.878C>T	p.A293V	0.993506
Dmd	X	NM_007868	splice	c.9629_splice	e67-3	0.108108
Dnah1	14	NM_001033668	silent	c.10677C>T	p.I3559I	0.246377
Dnah6	6	NM_001164669	silent	c.7413G>A	p.G2471G	0.60177
Dnaic2	11	NM_001034878	missense	c.293A>C	p.K98T	0.41791
Dnmt3l	10	NM_019448	missense	c.1153G>T	p.V385F	0.177778
Drd1a	13	NM_010076	missense	c.1459T>G	p.C487G	0.315789
Dtx2	5	NM_001256096	silent	c.18C>T	p.S6S	0.290323
Dtx2	5	NM_001256096	silent	c.30G>T	p.P10P	0.352941
Dym	18	NM_027727	missense	c.248C>G	p.S83C	0.27193
E2f7	10	NM_178609	missense	c.317A>T	p.K106M	0.683824
Egfl6	X	NM_019397	nonsense	c.1039A>T	p.K347*	1
Elk4	1	NM_007923	missense	c.178A>G	p.S60G	0.338235
Emc3	6	NM_175101	silent	c.378A>G	p.G126G	0.196721
Ets1	9	NM_011808	silent	c.1230C>T	p.Y410Y	0.308824

Disease Models & Mechanisms • Supplementary information

Exoc6	19	NM_175353	nonsense	c.2387T>A	p.L796*	0.5
F5	1	NM_007976	silent	c.504C>T	p.T168T	0.15
Fam120a	13	NM_0010332 68	silent	c.2185C>T	p.L729L	0.333333
Fam149a	8	NM_153535	missense	c.1129T>A	p.C377S	0.138889
Fam58b	11	NM_197989	missense	c.172T>C	p.Y58H	0.090909 1
Fam58b	11	NM_197989	silent	c.159G>A	p.P53P	0.101695
Fam58b	11	NM_197989	missense	c.152C>T	p.A51V	0.12
Fam58b	11	NM_197989	nonsense	c.145G>T	p.E49*	0.12
Fbn1	2	NM_007993	missense	c.1655A>G	p.D552G	0.30303
Foxh1	15	NM_007989	missense	c.53C>T	p.P18L	0.568966
Fras1	5	NM_175473	splice	c.8953_splice	e60-1	0.375
Frem3	8	NM_0011678 98	missense	c.4413G>T	p.E1471D	0.209302
Fsd1	17	NM_183178	splice	c.1381_splice	e13-2	0.238095
Fshr	17	NM_013523	missense	c.1985A>G	p.H662R	0.275362
Gbf1	19	NM_178930	frame_shift_d el	c.2376_2394del TGGGGAAGCACCCGTTA TT	p.P792fs	0.195122
Gkap1	13	NM_019832	missense	c.274G>A	p.V92I	0.412698
Gm101	1	NM_0011150 74	missense	c.1207G>A	p.A403T	0.317073
Gm362	X	NM_0011952 71	missense	c.923C>G	p.T308R	0.323529
Gm4922	10	NM_177706	missense	c.1263A>C	p.E421D	0.145833
Gm5088	14	NR_002862	missense	c.1330A>C	p.M444L	0.165854
Gm6607	9	NR_033622	missense	c.377G>T	p.R126M	0.647059
Gm6607	9	NR_033622	nonstop	c.375A>G	p.*125W	0.635714
Gm9705	17	NM_0011001 87	missense	c.1619T>G	p.V540G	0.717949
Gnat3	5	NM_0010811 43	missense	c.211G>T	p.A71S	0.466667
Gpsm1	2	NM_0011991 47	missense	c.937G>T	p.D313Y	0.214286
Gucy2c	6	NM_0011273 18	missense	c.1360C>A	p.L454M	0.36
H6pd	4	NM_173371	missense	c.482T>A	p.I161N	0.543478
Hist1h4f	13	NM_175655	missense	c.14G>T	p.G5V	0.331797
Hjurp	1	NM_198652	splice	c.163_splice	e3+2	0.113636
Hnrnpul2	19	NM_0010811 96	missense	c.1894C>G	p.P632A	0.180328
Hrasls5	19	NM_025731	missense	c.710A>G	p.K237R	0.510417
Hs6st2	X	NM_0010772 02	missense	c.1150T>C	p.W384R	0.243243
Htr1a	13	NM_008308	frame_shift_d el	c.4118delT	p.I1373fs	0.382353
Igsf9b	9	NM_0011297 87	missense	c.3662G>T	p.R1221L	0.238636
Il1r1	1	NM_008362	missense	c.1376G>A	p.R459K	0.157895

Disease Models & Mechanisms • Supplementary information

Il1rapl1	X	NM_001160403	missense	c.2056C>T	p.P686S	1
Itga6	2	NM_001277970	missense	c.2303A>G	p.D768G	0.265625
Itln1	1	NM_010584	silent	c.399C>T	p.D133D	0.0626632
Itsn1	16	NM_010587	missense	c.3781A>T	p.T1261S	0.25
Itsn1	16	NM_010587	silent	c.4746C>A	p.S1582S	0.526316
Itsn1	16	NM_010587	missense	c.4753G>A	p.A1585T	0.486486
Jag1	2	NM_013822	silent	c.2118C>T	p.D706D	0.275362
Kansl2	15	NM_133714	silent	c.705T>G	p.A235A	0.279762
Kcnb2	1	NM_001098528	missense	c.745C>A	p.L249I	0.789474
Kcnk15	2	NM_001030292	missense	c.412C>A	p.P138T	0.678571
Kcnq3	15	NM_152923	missense	c.1277G>T	p.G426V	0.323529
Kidins220	12	NM_001081378	missense	c.3101C>T	p.S1034L	0.428571
Kif9	9	NM_001163569	silent	c.321G>A	p.T107T	0.225806
Klf14	6	NM_001135093	missense	c.968C>A	p.S323Y	0.19375
Krt24	11	NM_029393	silent	c.837G>A	p.R279R	1
Lat2	5	NM_020044	missense	c.328G>T	p.D110Y	0.308824
Lcn6	2	NM_001276448	missense	c.527C>T	p.T176I	0.336449
Lcorl	5	NM_001163073	silent	c.1722G>A	p.K574K	0.363636
Lrrc43	5	NM_001033461	missense	c.1485G>T	p.M495I	0.333333
Madd	2	NM_001177720	missense	c.2278G>C	p.G760R	0.353535
Maged2	X	NM_001199246	missense	c.799G>A	p.E267K	0.559322
Map4k4	1	NM_001252200	frame_shift_ins	c.3754_3755insTAAA	p.L1252fs	0.159091
Men1	19	NM_001168488	silent	c.1038C>A	p.A346A	0.258242
Mgat1	11	NM_001110150	silent	c.1347T>G	p.P449P	0.0756303
Mical2	7	NM_001193305	missense	c.73T>G	p.C25G	0.673469
Mki67	7	NM_001081117	missense	c.2158T>G	p.S720A	0.206897
Mmgt2	11	NM_175002	missense	c.131G>T	p.S44I	0.233333
Mrgprb3	7	NM_207537	missense	c.439G>C	p.V147L	0.267606
Mroh1	15	NM_175457	missense	c.3162G>C	p.L1054F	0.185185
Ms4a6b	19	NM_027209	missense	c.544T>A	p.F182I	0.487179
Muc19	15	NM_207243	missense	c.2942G>C	p.W981S	0.254545
Myo18b	5	NM_028901	missense	c.6581A>C	p.H2194P	0.470588
Myo3a	2	NM_148413	frame_shift_ins	c.1591_1592insA	p.E531fs	0.185185

Disease Models & Mechanisms • Supplementary information

Myo5b	18	NM_201600	missense	c.4798C>A	p.L1600M	0.549451
Naa15	3	NM_053089	missense	c.72A>C	p.K24N	0.493506
Naa25	5	NM_172722	nonsense	c.1048G>T	p.E350*	0.269841
Nap1l2	X	NM_008671	missense	c.1657G>A	p.D553N	1
Nav2	7	NM_175272	missense	c.6454G>C	p.A2152P	0.235955
nb3	8	NM_173212	missense	c.919C>A	p.L307I	0.388889
Neto1	18	NM_144946	missense	c.503C>T	p.P168L	1
Nfasc	1	NM_182716	missense	c.904C>T	p.R302C	0.194444
Nhlrc4	17	NM_001039038	silent	c.222C>T	p.L74L	0.16129
Nit1	1	NM_012049	silent	c.165C>T	p.N55N	0.080292
Nom1	5	NM_001033457	splice	c.1094_splice	e2+3	0.130435
Npc2	12	NM_023409	missense	c.439A>T	p.I147F	0.23913
Npy5r	8	NM_016708	missense	c.420C>G	p.I140M	0.560606
Nyx	X	NM_173415	frame_shift_del	c.768delG	p.L256fs	0.238095
Obscn	11	NM_001171512	frame_shift_ins	c.12093_12094insC	p.K4032fs	0.146789
Olfml1	7	NM_172907	nonsense	c.184G>T	p.E62*	0.222222
Olfr1082	2	NM_207674	missense	c.484A>C	p.N162H	0.294479
Olfr1188	2	NM_146919	missense	c.653C>T	p.S218F	0.736111
Olfr1273-ps	2	NM_146975	missense	c.64A>C	p.K22Q	0.662162
Olfr1280	2	NM_146908	missense	c.528C>A	p.S176R	0.304478
Olfr1408	1	NM_146764	missense	c.484G>T	p.A162S	0.270777
Olfr1513	14	NM_001012269	silent	c.51C>T	p.V17V	0.297101
Olfr165	16	NM_146466	silent	c.276C>A	p.S92S	0.325
Olfr203	16	NM_146486	missense	c.419G>A	p.C140Y	0.534091
Olfr345	2	NM_146945	missense	c.32A>G	p.E11G	0.339506
Olfr426	1	NM_001206926	missense	c.679A>T	p.I227F	0.277778
Olfr493	7	NM_146310	missense	c.848T>C	p.V283A	0.336449
Olfr646	7	NM_147056	silent	c.894G>T	p.R298R	0.242424
Olfr666	7	NM_147096	missense	c.217G>A	p.V73I	0.343284
Olfr818	10	NM_146777	frame_shift_del	c.706delT	p.S236fs	0.15625
Olfr90	17	NM_146477	silent	c.153T>A	p.R51R	0.26087
Olfr916	9	NM_146784	missense	c.619A>G	p.I207V	0.243243
Olfr996	2	NM_146437	missense	c.277A>C	p.T93P	0.328244
Pcdha8	18	NM_201243	nonsense	c.1476C>G	p.Y492*	0.197802
Pcnxl3	19	NM_144868	silent	c.4875C>T	p.D1625D	0.261905
Pde1a	2	NM_016744	missense	c.1102A>C	p.S368R	0.339286
Phc2	4	NM_001195130	missense	c.2218G>A	p.E740K	0.53125
Phldb1	9	NM_153537	splice	c.2892_splice	e13+2	0.283019
Phldb1	9	NM_153537	missense	c.515C>T	p.T172I	0.72
Pkp4	2	NM_026361	missense	c.170T>A	p.V57E	0.382979

Disease Models & Mechanisms • Supplementary information

Plcl2	17	NM_013880	missense	c.1387G>T	p.A463S	0.178571
Pm20d1	1	NM_178079	missense	c.1165C>G	p.L389V	0.724638
Podnl1	8	NM_001013384	missense	c.716A>G	p.Y239C	0.14433
Pole	5	NM_011132	silent	c.4707C>A	p.R1569R	0.387097
Ppp1r3a	6	NM_080464	missense	c.404C>A	p.A135D	0.350365
Ppp1r3b	8	NM_177741	silent	c.435G>T	p.L145L	0.165049
Prb1	6	NM_198669	nonstop	c.1513T>G	p.*505E	0.190616
Prdm9	17	NM_144809	silent	c.276T>C	p.S92S	0.230769
Prickle2	6	NM_001081146	silent	c.2496C>T	p.C832C	0.19469
Prkaa2	4	NM_178143	missense	c.671A>G	p.K224R	0.289941
Prkcsh	9	NM_008925	missense	c.1156A>T	p.S386C	0.262032
Prpf19	19	NM_001253843	missense	c.767T>G	p.I256S	0.405797
Prpf4	4	NM_027297	splice	c.1373_splice	e14-2	0.227273
Prune2	19	NM_181348	missense	c.8829G>A	p.M2943I	0.276596
Ptgs2	1	NM_011198	missense	c.1138A>G	p.T380A	0.215385
Ptprj	2	NM_008982	missense	c.950T>A	p.V317E	0.8
Qpctl	7	NM_026111	missense	c.193C>T	p.R65W	0.666667
Raph1	1	NM_001045513	missense	c.1322T>C	p.V441A	0.18
Rasgrp2	19	NM_011242	silent	c.978G>T	p.R326R	0.228972
Rassf10	7	NM_175279	silent	c.2817A>G	p.K939K	0.136364
Rbm28	6	NM_133925	missense	c.1156G>T	p.A386S	0.4375
Reps1	10	NM_009048	splice	c.474_splice	e3+3_+23	0.224138
Rps6kc1	1	NM_178775	missense	c.2659T>A	p.S887T	0.751678
Scn2a1	2	NM_001099298	missense	c.443T>A	p.M148K	0.358025
Scn2a1	2	NM_001099298	silent	c.4071G>C	p.V1357V	0.773585
Scn9a	2	NM_018852	silent	c.897G>A	p.V299V	0.650485
Sec23a	12	NM_009147	silent	c.24C>T	p.I8I	0.28
Sema3g	14	NM_001025379	missense	c.1984T>C	p.F662L	0.126722
Shroom3	5	NM_015756	missense	c.521T>G	p.V174G	0.515152
Sirpb1a	3	NM_001002898	nonsense	c.615T>G	p.Y205*	0.166667
Sis	3	NM_001081137	missense	c.3902C>A	p.A1301D	0.5
Sis	3	NM_001081137	nonsense	c.1467T>G	p.Y489*	0.524476
Slc12a8	16	NM_134251	missense	c.1745G>A	p.C582Y	0.212766
Slc22a14	9	NM_001037749	missense	c.348C>G	p.N116K	0.606742
Slc25a2	18	NM_001159275	silent	c.1041A>G	p.E347E	0.315789
Slc33a1	3	NM_001272035	missense	c.46T>A	p.S16T	0.538462

Disease Models & Mechanisms • Supplementary information

Slc46a3	5	NM_027872	missense	c.430T>G	p.Y144D	0.0967742
Slc7a8	14	NM_016972	silent	c.753T>C	p.N251N	0.409091
Smarcd3	5	NM_025891	missense	c.1312G>A	p.A438T	0.259843
Smchd1	17	NM_028887	missense	c.38C>G	p.S13W	0.28125
Snx13	12	NM_001014973	missense	c.352C>A	p.Q118K	0.204082
Sox8	17	NM_011447	missense	c.1033C>G	p.P345A	0.713178
Sp100	1	NM_013673	nonsense	c.1408C>T	p.R470*	0.128205
Spatc1l	10	NM_029661	missense	c.70C>T	p.L24F	0.657895
Speer3	5	NM_027650	silent	c.738G>A	p.Q246Q	0.333333
Srsf6	2	NM_026499	nonsense	c.746C>A	p.S249*	0.288194
Ssr3	3	NM_026155	missense	c.416T>G	p.F139C	0.333333
Stab2	10	NM_138673	missense	c.6010G>A	p.A2004T	0.690909
Stc2	11	NM_011491	silent	c.882C>T	p.I294I	1
Suox	10	NM_173733	missense	c.1036G>A	p.E346K	0.394737
Svs1	6	NM_172888	nonsense	c.2324C>A	p.S775*	0.213904
Sympk	7	NM_026605	nonsense	c.2968G>T	p.E990*	0.661017
Syne1	10	NM_001079686	silent	c.6939C>T	p.T2313T	0.322581
Tas2r129	6	NM_207029	silent	c.666T>C	p.D222D	0.219101
Thrap3	4	NM_146153	silent	c.2799C>T	p.D933D	0.21875
Thsd4	9	NM_001040426	missense	c.2882C>A	p.S961Y	0.125
Tll2	19	NM_011904	missense	c.1158C>G	p.I386M	0.439716
Tmem108	9	NM_178638	silent	c.1161G>T	p.R387R	0.681818
Tmem184b	15	NM_001253819	silent	c.477C>T	p.C159C	0.230769
Tmem63b	17	NM_198167	missense	c.1424T>A	p.I475N	0.220183
Tnik	3	NM_001163009	missense	c.1243C>T	p.L415F	0.428571
Trhde	10	NM_146241	missense	c.382G>A	p.E128K	0.634146
Triobp	15	NM_001039156	silent	c.2109C>T	p.Y703Y	0.296992
Trp53	11	NM_011640	missense	c.823C>T	p.P275S	1
Trpm2	10	NM_138301	missense	c.1099A>C	p.T367P	0.577465
Trpm8	1	NM_134252	missense	c.3200T>G	p.I1067S	0.30303
Tshz3	7	NM_172298	silent	c.1902G>A	p.K634K	0.605263
Tsr1	11	NM_177325	silent	c.1773T>A	p.S591S	0.097561
Txnl1	18	NM_016792	missense	c.479A>C	p.D160A	0.333333
Ugp2	11	NM_139297	missense	c.479T>A	p.V160D	1
Unc5d	8	NM_153135	silent	c.2391C>A	p.R797R	0.328671
Uts2r	11	NM_145440	missense	c.163C>A	p.P55T	0.268519
Vldlr	19	NM_013703	missense	c.605C>T	p.T202I	0.47619
Wdr45	X	NM_172372	missense	c.294G>C	p.E98D	0.333333
Wdr90	17	NM_001163766	missense	c.4897T>C	p.S1633P	0.162162

Disease Models & Mechanisms • Supplementary information

Whamm	7	NM_001004185	missense	c.980C>T	p.P327L	0.75
Wwp1	4	NM_177327	nonsense	c.490G>T	p.G164*	0.435897
Ythdc2	18	NM_001163013	silent	c.3577C>A	p.R1193R	1
Zbtb12	17	NM_198886	missense	c.1571G>A	p.C524Y	0.407407
Zcchc11	4	NM_175472	missense	c.4442C>A	p.P1481H	0.777778
Zfat	15	NM_001145888	missense	c.2774G>A	p.R925H	0.348485
Zfp623	15	NM_030199	silent	c.525G>A	p.T175T	0.34375
Zp1	19	NM_009580	missense	c.1819G>A	p.V607M	0.397727
1700017D01Rik	19	NM_027058	missense	c.419C>T	p.A140V	0.533333
1810033B17Rik	8	NM_026985	missense	c.512A>C	p.E171A	0.590909
2310042D19Rik	4	NM_172417	silent	c.918T>C	p.P306P	0.482143
2610028H24Rik	10	NM_029816	missense	c.66G>T	p.M22I	0.140351
2700097O09Rik	12	NM_028314	missense	c.895A>G	p.K299E	0.32
4933409G03Rik	2	NM_177651	missense	c.137A>G	p.N46S	0.344828
A430078G23Rik	8	NM_001033378	missense	c.1010A>G	p.Q337R	0.272727
A530016L24Rik	12	NM_177039	silent	c.306C>T	p.G102G	0.588235

Disease Models & Mechanisms • Supplementary information

Table S2. Mutated coding genes in UN-SCC680 cells

Name	Chr	Transcript	Mutation type	DNA change	Protein change	Allelic fraction
Abca8b	11	NM_013851	silent	c.4554G>A	p.K1518K	0.506024
Abcb1a	5	NM_011076	silent	c.3741C>A	p.V1247V	0.093167 7
Abcb1b	5	NM_011075	missense	c.3677T>C	p.I1226T	0.57265
Abcc9	6	NM_021041	missense	c.3290C>T	p.T1097M	0.62
Acbd4	11	NM_025988	missense	c.569C>T	p.T190I	0.536232
Actr10	12	NM_019785	missense	c.833A>C	p.K278T	0.111111
Adam9	8	NM_001270996	silent	c.1056C>T	p.N352N	0.44086
Adamts3	5	NM_177872	missense	c.736C>T	p.R246W	0.103448
Adcy2	13	NM_153534	missense	c.377A>T	p.Y126F	0.425926
Adcy8	15	NM_009623	silent	c.2604C>G	p.A868A	0.077844 3
Akap12	10	NM_031185	missense	c.884G>C	p.R295P	0.624138
Akap8	17	NM_019774	missense	c.659C>T	p.S220F	0.396226
Akt3	1	NM_011785	missense	c.722G>C	p.R241P	0.25
Aldh1a3	7	NM_053080	missense	c.784G>C	p.G262R	0.464912
Alms1	6	NM_145223	missense	c.6033G>T	p.E2011D	0.605556
Alpk3	7	NM_054085	missense	c.3647T>G	p.V1216G	0.7
Angpt2	8	NM_007426	missense	c.407C>T	p.A136V	0.157143
Ank	15	NM_020332	missense	c.1032G>T	p.W344C	0.089219 3
Ankrd32	13	NM_134071	silent	c.2250A>G	p.K750K	0.272727
Anks1b	10	NM_001128086	silent	c.1482C>T	p.H494H	0.587719
Anxa13	15	NM_027211	missense	c.173A>C	p.K58T	0.052884 6
Apbb2	5	NM_009686	missense	c.1691C>T	p.S564F	0.425926
Aplnr	2	NM_011784	silent	c.3501G>T	p.A1167A	0.085106 4
Arl4d	11	NM_025404	missense	c.1245A>T	p.L415F	0.115385
Atp1a4	1	NM_013734	missense	c.2410A>C	p.I804L	0.333333
B230118H07Ri k	2	NM_026592	missense	c.66T>A	p.D22E	0.706897
B4galt2	4	NM_001253381	nonsense	c.1078G>T	p.G360*	0.229167
BC049635	4	NM_177785	silent	c.642C>T	p.F214F	0.421429
BC051628	2	NM_199312	missense	c.520G>C	p.D174H	0.628571
Bcl2l13	6	NM_153516	missense	c.662T>A	p.I221N	0.362832
Bdp1	13	NM_001081061	missense	c.4744C>G	p.P1582A	0.2
Bmi1	2	NM_007552	missense	c.811C>G	p.P271A	0.313514
C530008M17R ik	5	NM_001163793	silent	c.3207C>T	p.S1069S	0.534884
Cabp4	19	NM_144532	silent	c.372T>A	p.P124P	0.131313
Cap2	13	NM_026056	silent	c.399C>G	p.L133L	0.459119
Ccnb1	13	NM_172301	splice	c.537_splice	e5+1	0.42623
Cdh23	10	NM_001252635	missense	c.6338G>A	p.R2113H	0.216216

Cdh23	10	NM_023370	missense	c.4639C>A	p.P1547T	0.594203
Cdyl	13	NM_009881	frame_shift_del	c.1698delT	p.C566fs	0.413043
Cep78	19	NM_198019	missense	c.1859T>C	p.V620A	0.586667
Ces4a	8	NM_146213	missense	c.907G>A	p.V303I	0.35
Chst10	1	NM_145142	missense	c.270C>G	p.D90E	0.297872
Ckap2	8	NM_001004140	missense	c.602C>T	p.T201I	0.106195
Clec12a	6	NM_177686	missense	c.320C>A	p.S107Y	0.418605
Cnot3	7	NM_146176	missense	c.611A>G	p.K204R	0.116071
Col4a1	8	NM_009931	missense	c.263G>T	p.G88V	0.611111
Col5a1	2	NM_015734	missense	c.3107G>T	p.G1036V	0.142857
Cops7a	6	NM_001164089	missense	c.511G>A	p.A171T	0.52381
Cpsf1	15	NM_001164173	silent	c.867G>T	p.L289L	0.0642202
Csf1r	18	NM_001037859	missense	c.2852G>A	p.S951N	0.538462
Csf2rb	15	NM_007780	missense	c.706G>A	p.V236I	0.229358
Csf2rb2	15	NM_007781	missense	c.709G>A	p.V237I	0.823529
Csmd3	15	NM_001081391	missense	c.12C>G	p.S4R	0.118919
Ctsll3	13	NM_027344	silent	c.963G>A	p.G321G	0.46875
Cyp2c29	19	NM_007815	missense	c.724A>C	p.S242R	0.0897436
Dcaf12l2	X	NM_175539	missense	c.1301C>A	p.S434Y	1
Dct	14	NM_010024	silent	c.927A>G	p.R309R	0.258621
Dgke	11	NM_019505	missense	c.1435G>T	p.V479F	0.429688
Dnah6	6	NM_001164669	missense	c.8914C>T	p.R2972W	0.368421
Dpf1	7	NM_013874	silent	c.1122C>T	p.L374L	0.553672
Dpp4	2	NM_010074	missense	c.989C>T	p.T330M	0.245283
Dstyk	1	NM_172516	missense	c.1976G>A	p.G659D	0.711111
Dusp11	6	NM_028099	silent	c.945C>T	p.Y315Y	0.660714
E2f1	2	NM_007891	splice	c.826_splice	e7-3_-4	0.0853785
Eef2k	7	NM_001267711	missense	c.398C>A	p.A133E	0.168831
Eif4enif1	11	NM_023743	missense	c.30T>G	p.N10K	0.276316
Elmo1	13	NR_038122	missense	c.208T>A	p.C70S	0.444444
Emcn	3	NM_001163522	missense	c.215T>A	p.V72E	0.411765
En2	5	NM_010134	missense	c.649C>A	p.P217T	0.135135
Enam	5	NM_017468	nonsense	c.2980G>T	p.E994*	0.209302
Enam	5	NM_017468	missense	c.1098G>T	p.W366C	0.571429
Ern2	7	NM_012016	nonsense	c.937G>T	p.G313*	0.265193
Fam83a	15	NM_173862	missense	c.612G>A	p.M204I	0.0671642
Fasn	11	NM_007988	missense	c.1742C>T	p.S581F	0.0652174
Fat2	11	NM_001029988	silent	c.8700C>T	p.D2900D	0.486726
Fat3	9	NM_001080814	silent	c.1029A>G	p.K343K	0.721429
Fat4	3	NM_183221	silent	c.7113T>G	p.T2371T	0.228261
Fbn1	2	NM_007993	missense	c.980G>T	p.R327I	0.652174

Disease Models & Mechanisms • Supplementary information

Fbn2	18	NM_010181	missense	c.4309G>C	p.G1437R	0.423077
Fbxo15	18	NM_015798	missense	c.452G>A	p.S151N	0.590909
Fbxw13	9	NM_177598	splice	c.293_splice	e5-1	0.336918
Fbxw20	9	NM_001008428	silent	c.27A>G	p.P9P	0.649077
Fgb	3	NM_181849	missense	c.83C>T	p.T28M	0.265306
Fmo9	1	NM_172844	missense	c.529T>G	p.Y177D	0.775
Foxp2	6	NM_053242	missense	c.1489T>A	p.Y497N	0.126761
Galnt12	4	NM_172693	silent	c.369G>A	p.V123V	0.219178
Gbp4	5	NM_001256005	missense	c.296T>A	p.L99Q	0.108108
Gcc2	10	NM_027375	nonsense	c.2509G>T	p.E837*	0.164384
Gjc2	11	NM_080454	silent	c.498C>A	p.G166G	0.288889
Gm5114	7	NM_177890	missense	c.2079C>G	p.H693Q	0.178571
Gm9268	7	NM_001105061	missense	c.1155C>A	p.D385E	0.189258
Grin3a	4	NM_001276355	missense	c.1126G>T	p.A376S	0.153846
Gtf2h4	17	NM_010364	missense	c.689A>C	p.D230A	0.145833
H2-M10.5	17	NM_177637	missense	c.145G>A	p.G49S	0.475524
Haghl	17	NM_026897	missense	c.527C>A	p.T176K	0.5
Hao1	2	NM_010403	silent	c.987T>G	p.V329V	0.298969
Hdac8	X	NM_027382	nonsense	c.616G>T	p.G206*	0.468085
Hdac9	12	NM_001271386	missense	c.3049G>T	p.A1017S	0.708333
Hist1h1d	13	NM_145713	missense	c.1089T>A	p.N363K	0.081081 1
Hoxa2	6	NM_010451	missense	c.527T>C	p.L176P	0.11828
Hrh1	6	NM_001252642	frame_shift_d el	c.1476delA	p.S492fs	0.128205
Htr1f	16	NM_008310	missense	c.76A>G	p.T26A	0.387097
Icam5	9	NM_008319	missense	c.98C>A	p.P33H	0.072992 7
Ift88	14	NM_009376	missense	c.623C>T	p.S208F	0.350649
Ints1	5	NM_026748	silent	c.1582C>T	p.L528L	0.405405
Ints3	3	NM_145540	missense	c.1433C>G	p.P478R	0.25
Ip6k3	17	NM_173027	missense	c.535A>G	p.T179A	0.566038
Ipo8	6	NM_001081113	silent	c.2502G>T	p.R834R	0.345324
Irgm2	11	NM_019440	silent	c.1281C>T	p.P427P	0.089552 2
Isg20l2	3	NM_177663	missense	c.238G>A	p.E80K	0.148148
Itgax	7	NM_021334	missense	c.847A>C	p.I283L	0.565217
Jak3	8	NM_010589	missense	c.796G>T	p.G266W	0.241379
Kalrn	16	NM_177357	missense	c.6913G>A	p.D2305N	0.605263
Kcnj12	11	NM_001267593	missense	c.1973T>A	p.M658K	0.503876
Kcnt2	1	NM_001081027	missense	c.1792G>T	p.A598S	0.470588
Kif13b	14	NM_001081177	silent	c.4437C>T	p.H1479H	0.625
Kitl	10	NM_013598	missense	c.500C>T	p.S167L	0.572519
Kmt2a	9	NM_001081049	missense	c.1109C>T	p.A370V	0.45
Kras	6	NM_021284	missense	c.175G>A	p.A59T	0.291139
Krt33a	11	NM_027983	missense	c.1021G>A	p.V341M	0.143312

Krt78	15	NM_212487	missense	c.1113G>T	p.E371D	0.0963855
Krtap10-10	10	NM_001024709	missense	c.986G>C	p.C329S	0.164286
Lap3	5	NM_024434	nonsense	c.1205C>A	p.S402*	0.47561
Lhfpl5	17	NM_026571	silent	c.9G>A	p.K3K	0.546099
Lingo3	10	NM_001013758	missense	c.1312A>C	p.T438P	0.1875
Lrit1	14	NM_146245	silent	c.501G>A	p.L167L	0.654762
Lrrc30	17	NM_001033340	missense	c.1051A>C	p.S351R	0.176471
Lrrn3	12	NM_010733	missense	c.706G>A	p.A236T	0.674419
Ltb	17	NM_008518	silent	c.54C>T	p.C18C	0.23741
Map3k5	10	NM_008580	missense	c.1277T>G	p.F426C	0.517647
Mboat7	7	NM_029934	silent	c.1287C>A	p.I429I	0.157895
Mcc	18	NM_001085373	missense	c.2692G>A	p.A898T	0.223404
Mos	4	NM_020021	missense	c.315T>G	p.C105W	0.43913
Mphosph6	8	NM_026758	splice	c.354_splice	e6-3	0.125
Mphosph8	14	NM_023773	missense	c.236G>T	p.R79L	0.75
Mrpl17	7	NM_025301	silent	c.201C>T	p.D67D	0.54023
Msx2	13	NM_013601	missense	c.484C>T	p.R162C	0.302158
Muc6	7	NM_181729	silent	c.3279C>T	p.T1093T	0.297297
N4bp2	5	NM_001024917	missense	c.220T>G	p.L74V	0.504399
Neb	2	NM_010889	missense	c.1698G>T	p.K566N	0.240741
Neb	2	NM_010889	missense	c.1391G>T	p.G464V	0.357576
Nefh	11	NM_010904	missense	c.1192G>T	p.A398S	0.128571
Nlrp3	11	NM_145827	silent	c.2727G>A	p.Q909Q	0.197917
Nop56	2	NM_024193	missense	c.682T>G	p.L228V	0.351351
Npc1l1	11	NM_207242	silent	c.888A>G	p.V296V	0.415094
Npr1	3	NM_008727	missense	c.1526A>G	p.E509G	0.166667
Nrip1	16	NM_173440	nonsense	c.2824C>T	p.Q942*	0.333333
Nsun7	5	NM_027602	missense	c.1595C>G	p.A532G	0.238462
Nt5c1b	12	NM_027588	silent	c.168C>T	p.Y56Y	0.816327
Nvl	1	NM_026171	splice	c.2364_splice	e22-1	0.0833333
Nyap1	5	NM_175521	missense	c.488C>T	p.P163L	0.466102
Ocstamp	2	NM_029021	silent	c.388C>T	p.L130L	0.233766
Olfr1058	2	NM_146391	missense	c.290G>A	p.C97Y	0.309091
Olfr114	17	NM_146287	missense	c.55G>A	p.D19N	0.489914
Olfr1276	2	NM_146395	missense	c.232G>A	p.V78I	0.248062
Olfr1302	2	NM_146889	silent	c.756C>A	p.I252I	0.321608
Olfr1314	2	NM_146450	missense	c.794C>A	p.T265K	0.246914
Olfr1413	1	NM_147037	missense	c.37C>T	p.L13F	0.64557
Olfr1442	19	NM_146697	silent	c.900G>A	p.K300K	0.33913
Olfr1477	19	NM_146696	missense	c.585C>A	p.F195L	0.185252
Olfr153	2	NM_206823	missense	c.416A>C	p.K139T	0.378049
Olfr170	16	NM_146957	silent	c.456G>A	p.G152G	0.3
Olfr220	1	NM_207694	missense	c.713G>A	p.G238D	0.286432

Disease Models & Mechanisms • Supplementary information

Olfr235	19	NM_146686	missense	c.424T>A	p.C142S	0.606218
Olfr446	6	NM_146295	missense	c.308C>A	p.A103D	0.228571
Olfr476	7	NM_146924	missense	c.544C>G	p.P182A	0.469136
Olfr484	7	NM_146499	silent	c.24C>T	p.N8N	0.532258
Olfr508	7	NM_146773	nonsense	c.381C>A	p.C127*	0.085889 6
Olfr727	14	NM_146319	missense	c.436G>C	p.V146L	0.138686
Olfr894	9	NM_146868	missense	c.779G>T	p.W260L	0.26749
Oma1	4	NM_025909	frame_shift_d el	c.1037delT	p.L346fs	0.948148
Otp	13	NM_011021	missense	c.154G>A	p.V52I	0.455497
P2rx3	2	NM_145526	missense	c.166A>C	p.I56L	0.25
Pafah1b1	11	NM_013625	missense	c.737G>A	p.G246D	0.448052
Palm2	4	NM_172868	silent	c.534T>C	p.Y178Y	0.448276
Pappa	4	NM_021362	missense	c.1107G>C	p.W369C	0.182927
Pcdh9	14	NM_001081377	missense	c.3148C>T	p.R1050C	0.282051
Pcdhb1	18	NM_053126	missense	c.451C>T	p.R151C	0.071428 6
Pcdhb10	18	NM_053135	missense	c.2528G>A	p.G843D	0.520833
Pcdhb13	18	NM_053138	missense	c.1721C>T	p.A574V	0.049900 2
Pcdhb19	18	NM_053144	silent	c.3321C>A	p.S1107S	0.085714 3
Pcdhb9	18	NM_053134	missense	c.2285A>T	p.H762L	0.507614
Pcdhgb7	18	NM_033579	silent	c.1977C>A	p.L659L	0.174757
Pdzd2	15	NM_001081064	missense	c.861C>A	p.D287E	0.243478
Pdzd9	7	NM_001040136	splice	c.401_splice	e4+3_+7	0.191489
Pgd	4	NM_001081274	missense	c.1367A>C	p.E456A	0.395349
Pgr	9	NM_008829	silent	c.105T>G	p.G35G	0.272727
Pitpnm2	5	NM_011256	silent	c.883C>A	p.R295R	0.479592
Ppp1r13l	7	NM_001010836	missense	c.1975G>T	p.A659S	0.301075
Pramef8	4	NM_172877	missense	c.502C>A	p.Q168K	1
Prom2	2	NM_138750	silent	c.195C>T	p.G65G	0.131579
Prp2	6	NM_031499	missense	c.775G>A	p.G259S	0.080645 2
Prr15	6	NM_030024	missense	c.867C>A	p.N289K	0.163636
Psd2	18	NM_028707	missense	c.394G>A	p.D132N	0.5
Psmc3ip	11	NM_008949	missense	c.140T>C	p.V47A	0.185567
Ptpn23	9	NM_001081043	missense	c.2182C>G	p.P728A	0.30303
Qdpr	5	NM_024236	splice	c.427_splice	e5+2	0.073529 4
Rab6a	7	NM_001163663	missense	c.518C>A	p.A173D	0.276923
Ran	5	NM_009391	silent	c.150C>T	p.L50L	0.116129
Ran	5	NM_009391	missense	c.146C>T	p.P49L	0.117241
Rap2b	3	NM_028712	missense	c.3395A>T	p.N1132I	0.122807
Rasef	4	NM_001017427	missense	c.809C>A	p.T270K	0.210526
Rasl10a	11	NM_145216	missense	c.85T>G	p.Y29D	0.247706

Disease Models & Mechanisms • Supplementary information

Rb1	14	NM_009029	splice	c.1197_splice	e13+2	0.714286
Rcc1	4	NM_001197082	silent	c.1122C>T	p.S374S	0.342105
Rgs1	1	NM_015811	missense	c.562C>T	p.P188S	0.58
Rgs12	5	NM_173402	missense	c.917A>C	p.D306A	0.165217
Rhof	5	NM_175092	missense	c.203_204delins AT	p.T68N	0.401535
Rnase4	14	NM_021472	silent	c.513C>T	p.D171D	0.486486
Ror2	13	NM_013846	splice	c.464_splice	e5-1	0.439024
Rpl3l	17	NM_001163945	missense	c.103G>A	p.D35N	0.588235
Rundc1	11	NM_172566	missense	c.617A>T	p.E206V	0.555556
Samd3	10	NM_001115154	missense	c.1056G>T	p.K352N	0.52381
Sapcd1	17	NM_023893	silent	c.180G>A	p.E60E	0.52
Scg2	1	NM_009129	silent	c.1518C>A	p.T506T	0.65
Sel1l3	5	NM_172710	missense	c.1976A>G	p.Y659C	0.567568
Serpinb6d	13	NM_001076790	missense	c.811G>T	p.V271F	0.259615
Setbp1	18	NM_053099	missense	c.3670A>T	p.T1224S	0.514286
Sfmbt2	2	NM_001198808	missense	c.2110G>A	p.V704I	0.723404
Sh2d3c	2	NM_013781	missense	c.1729T>C	p.S577P	0.304636
Sim2	16	NM_011377	silent	c.1488T>C	p.S496S	0.301887
Sipa1l2	8	NM_001081337	missense	c.1370C>G	p.S457C	0.081081 1
Slc10a4	5	NM_173403	missense	c.1067T>C	p.F356S	0.060126 6
Slc26a3	12	NM_021353	missense	c.1024G>A	p.V342I	0.231884
Slc41a3	6	NM_027868	nonsense	c.923C>A	p.S308*	0.094339 6
Slc5a4a	10	NM_133184	splice	c.1449_splice	e12+1	0.429907
Slc6a20b	9	NM_011731	silent	c.1416C>G	p.P472P	0.076923 1
Slc9a9	9	NM_177909	splice	c.1707_splice	e15+1	0.333333
Slit1	19	NM_015748	silent	c.900C>A	p.L300L	0.35443
Sox30	11	NM_173384	nonsense	c.994G>T	p.E332*	0.525253
Speg	1	NM_007463	silent	c.6369C>A	p.P2123P	0.266667
Spint1	2	NM_016907	nonsense	c.786C>A	p.Y262*	0.673077
St8sia1	6	NM_011374	silent	c.942C>T	p.V314V	0.214286
Stap1	5	NM_019992	silent	c.612G>A	p.L204L	0.4
Sult1d1	5	NM_016771	missense	c.430C>A	p.Q144K	0.463415
Swt1	1	NM_025819	missense	c.2390C>A	p.S797Y	0.16129
Synj1	16	NM_001164483	silent	c.531C>T	p.V177V	0.661765
Syt2	1	NM_009307	silent	c.771C>A	p.I257I	0.316901
Taf2	15	NM_001081288	silent	c.1287T>G	p.S429S	0.28
Tbc1d10c	19	NM_178650	missense	c.556C>A	p.L186I	0.568627
Tcl1b1	12	NM_013773	splice	c.307_splice	e3-3	0.244828
Tcp11l2	10	NM_146008	silent	c.162A>T	p.T54T	0.559701
Tex21	12	NM_019784	missense	c.25A>G	p.S9G	0.288889
Thsd7a	6	NM_001164805	missense	c.4736G>T	p.G1579V	0.401709

Disease Models & Mechanisms • Supplementary information

Tmem150a	6	NM_144916	missense	c.34T>A	p.S12T	0.379902
Tmem196	12	NM_001160385	frame_shift_del	c.184delA	p.K62fs	0.24812
Tmem26	10	NM_177794	silent	c.949C>A	p.R317R	0.173469
Tmem92	11	NM_001034896	nonsense	c.64G>T	p.G22*	0.131579
Tmprss6	15	NM_027902	nonsense	c.936G>A	p.W312*	0.142857
Tnfaip8l1	17	NM_025566	missense	c.479G>T	p.R160L	0.181818
Tnn	1	NM_177839	missense	c.2786T>C	p.V929A	0.208333
Tns1	1	NM_027884	silent	c.2337C>T	p.H779H	0.62069
Tpcn1	5	NM_145853	nonsense	c.371C>A	p.S124*	0.541667
Tpr	1	NM_133780	silent	c.7173C>T	p.F2391F	0.330986
Tpt1	14	NM_009429	silent	c.441T>G	p.A147A	0.604651
Trank1	9	NM_001164659	silent	c.6613C>T	p.L2205L	0.290323
Trhde	10	NM_146241	missense	c.2155G>A	p.A719T	0.157895
Tsnaxip1	8	NM_024445	missense	c.590A>G	p.E197G	0.7
Ttll2	17	NM_001098267	missense	c.1258A>C	p.T420P	0.556818
Ube2o	11	NM_173755	missense	c.1048T>A	p.Y350N	0.587571
Ubl7	9	NM_001122873	missense	c.754C>T	p.R252C	0.44
Ugt2b35	5	NM_172881	nonsense	c.1192G>T	p.E398*	0.258503
Usp31	7	NM_001033173	missense	c.3605G>T	p.S1202I	0.285714
Vmn2r106	17	NM_001104568	missense	c.2206T>C	p.Y736H	0.0595238
Vmn2r3	3	NM_001104614	missense	c.1972T>C	p.W658R	0.283951
Vmn2r73	7	NM_001105186	silent	c.207C>A	p.P69P	0.275362
Vsx2	12	NM_007701	missense	c.807G>C	p.K269N	0.535714
Wdr52	16	NM_001033247	silent	c.5460G>A	p.R1820R	0.646688
Wipf2	11	NM_197940	silent	c.315G>A	p.E105E	0.363636
Wtap	17	NM_001113533	missense	c.750_751delinsAC	p.ST250_251RP	0.120639
Xylt1	7	NM_175645	missense	c.2161G>A	p.V721I	0.22973
Zbtb38	9	NM_175537	frame_shift_ins	c.3406_3407insA	p.I1136fs	0.155172
Zc3h6	2	NM_178404	missense	c.3389C>A	p.T1130K	0.681818
Zfhx3	8	NM_007496	missense	c.3234T>A	p.H1078Q	0.375
Zfp174	16	NM_001081217	missense	c.586G>A	p.V196I	0.259259
Zfp354c	11	NM_013922	silent	c.25C>A	p.R9R	0.242938
Zfp407	18	NM_001033341	missense	c.6152T>A	p.V2051D	0.482143
Zfp451	1	NM_133817	missense	c.380C>T	p.T127I	0.675676
Zfp786	6	NM_177882	missense	c.233A>T	p.D78V	0.328125
Zhx3	2	NM_177263	missense	c.1159C>A	p.L387I	0.454545
Zmynd8	2	NM_027230	missense	c.1101G>T	p.M367I	0.0930233
Zmynd8	2	NM_027230	silent	c.1074G>A	p.K358K	0.105263
1700001J03Rik	5	NM_001008547	missense	c.80G>A	p.R27H	0.217391
1700008O03Rik	7	NM_027049	missense	c.137C>A	p.S46Y	0.407407

Disease Models & Mechanisms • Supplementary information

1700012A03Rik	6	NM_029587	missense	c.217G>A	p.D73N	0.545455
2-Mar	1	NM_133684	silent	c.448C>T	p.L150L	0.214286
2610034M16Rik	17	NM_027001	missense	c.990C>A	p.N330K	0.22619
2810408A11Rik	11	NM_027419	missense	c.313A>G	p.T105A	0.625
2900026A02Rik	5	NM_172884	missense	c.763C>T	p.P255S	0.391304
4930483J18Rik	15	NR_015603	silent	c.462G>T	p.L154L	0.0851064
4930511M11Rik	5	NM_029141	missense	c.396C>A	p.F132L	0.24
5530401A14Rik	11	NR_038010	missense	c.94G>A	p.V32I	0.444444
9130019O22Rik	7	NM_030226	missense	c.314A>C	p.K105T	0.10828
9930013L23Rik	7	NM_030728	silent	c.3099C>T	p.A1033A	0.241758
A330076H08Rik	7	NR_015599	silent	c.90G>T	p.G30G	0.328767
AA792892	5	NM_178894	missense	c.62C>T	p.S21F	0.218182

Disease Models & Mechanisms • Supplementary information

Table S3. Top 100 differentially expressed genes in UN-SCC679 cells vs lung airway basal cells.

Name	Gene	Log2 fold change	Adj p-value
Arnt2	ENSMUSG00000015709	11.0620074	< 1e-16
Tmod4	ENSMUSG00000005628	10.8697962	< 1e-16
Mb	ENSMUSG00000018893	9.48403985	< 1e-16
Dnah1	ENSMUSG00000019027	9.46694953	< 1e-16
Serpinf1	ENSMUSG00000000753	9.3299703	< 1e-16
2210416O15Rik	ENSMUSG00000018378	9.18210183	< 1e-16
Angptl4	ENSMUSG00000002289	9.05863394	< 1e-16
Eno2	ENSMUSG00000004267	8.29265377	< 1e-16
Atp1a2	ENSMUSG00000007097	8.04111543	< 1e-16
Dhdh	ENSMUSG00000011382	7.90003648	< 1e-16
Relt	ENSMUSG00000008318	7.86287023	< 1e-16
Chrd	ENSMUSG00000006958	7.83379912	< 1e-16
Pkib	ENSMUSG00000019876	7.78371557	< 1e-16
Msh5	ENSMUSG00000007035	7.45023936	< 1e-16
Ttc25	ENSMUSG00000006784	7.07810055	< 1e-16
Apc2	ENSMUSG00000020135	6.94197954	< 1e-16
Plekho1	ENSMUSG00000015745	6.87018629	< 1e-16
Vwa7	ENSMUSG00000007030	6.63977884	< 1e-16
Hmg20b	ENSMUSG00000020232	6.57350545	< 1e-16
Dnah2	ENSMUSG00000005237	6.45455317	< 1e-16
Lamb1	ENSMUSG00000002900	6.42761631	< 1e-16
Pcsk4	ENSMUSG00000020131	6.41467084	< 1e-16
Slc12a5	ENSMUSG00000017740	6.19618629	< 1e-16
Rundc3a	ENSMUSG00000006575	6.10565229	< 1e-16
Slc5a6	ENSMUSG00000006641	6.03452115	< 1e-16
Slc25a22	ENSMUSG00000019082	6.03400876	< 1e-16
Nav1	ENSMUSG00000009418	5.9852981	< 1e-16
Neu1	ENSMUSG00000007038	5.7582909	< 1e-16
Atp5g1	ENSMUSG00000006057	5.63948235	< 1e-16
Sult2b1	ENSMUSG00000003271	5.60165717	< 1e-16
Mlxipl	ENSMUSG00000005373	5.50159784	< 1e-16
Yeats4	ENSMUSG00000020171	5.38914096	< 1e-16
Taf6l	ENSMUSG00000003680	5.31866434	< 1e-16
Dhx34	ENSMUSG00000006019	5.30781679	< 1e-16
Cyth2	ENSMUSG00000003269	5.24920865	< 1e-16
Zfp287	ENSMUSG00000005267	5.14478228	< 1e-16
Fam184b	ENSMUSG00000015879	5.10345832	< 1e-16
Rasa4	ENSMUSG00000004952	4.96693919	< 1e-16
Fblim1	ENSMUSG00000006219	4.96270914	< 1e-16
Rufy1	ENSMUSG00000020375	4.90412027	< 1e-16

Dennd2c	ENSMUSG00000007379	4.9038307	< 1e-16
Evi5l	ENSMUSG00000011832	4.8847123	< 1e-16
Ppfia3	ENSMUSG00000003863	4.82184075	< 1e-16
Armc6	ENSMUSG00000002343	4.81718049	< 1e-16
Dyrk3	ENSMUSG00000016526	4.79401568	< 1e-16
G3bp1	ENSMUSG00000018583	4.77329742	< 1e-16
Snrpd1	ENSMUSG00000002477	4.64426595	< 1e-16
Snd1	ENSMUSG00000001424	4.58347156	< 1e-16
Csf1	ENSMUSG00000014599	4.55864729	< 1e-16
Capza2	ENSMUSG00000015733	4.52801991	< 1e-16
Slc7a7	ENSMUSG00000000958	4.4930873	< 1e-16
Slc25a13	ENSMUSG00000015112	4.47813393	< 1e-16
Impdh1	ENSMUSG00000003500	4.47331387	< 1e-16
Nkiras2	ENSMUSG00000017837	4.39726769	< 1e-16
Etv5	ENSMUSG00000013089	4.20675162	< 1e-16
Smo	ENSMUSG00000001761	4.19704028	< 1e-16
Vps50	ENSMUSG00000001376	4.18363994	< 1e-16
Atad5	ENSMUSG00000017550	4.07505785	< 1e-16
Tpra1	ENSMUSG00000002871	4.03374748	< 1e-16
Tnk1	ENSMUSG00000001583	4.02436232	< 1e-16
Gtf2h4	ENSMUSG00000001524	4.0240347	< 1e-16
Zfp959	ENSMUSG00000003198	3.94384642	< 1e-16
Sf3a2	ENSMUSG00000020211	3.91560916	< 1e-16
Met	ENSMUSG00000009376	3.87874932	< 1e-16
Ptpn6	ENSMUSG00000004266	3.84290539	< 1e-16
Dgke	ENSMUSG00000000276	3.80678444	< 1e-16
Cacnb3	ENSMUSG00000003352	3.80529652	< 1e-16
Zfp385a	ENSMUSG00000000552	3.65874456	< 1e-16
Map1s	ENSMUSG00000019261	3.53339114	< 1e-16
Ccar1	ENSMUSG00000020074	3.53191376	< 1e-16
Fgd6	ENSMUSG00000020021	3.50288373	< 1e-16
Slc5a8	ENSMUSG00000020062	3.47862885	< 1e-16
Cav1	ENSMUSG00000007655	3.45514074	< 1e-16
Tnpo3	ENSMUSG00000012535	3.44830198	< 1e-16
Mrpl4	ENSMUSG00000003299	3.44700769	< 1e-16
Lnx2	ENSMUSG00000016520	3.42992571	< 1e-16
Cacfd1	ENSMUSG00000015488	3.3871953	< 1e-16
Dhx40	ENSMUSG00000018425	3.38313608	< 1e-16
Ankrd13d	ENSMUSG00000005986	3.36682094	< 1e-16
Dbf4	ENSMUSG00000002297	3.33106415	< 1e-16
Rbsn	ENSMUSG00000014550	3.32925014	< 1e-16
Gtf2ird2	ENSMUSG00000015942	3.28956608	< 1e-16
Mocs2	ENSMUSG00000015536	3.26040237	< 1e-16
Anxa6	ENSMUSG00000018340	3.26020574	< 1e-16

Disease Models & Mechanisms • Supplementary information

Prpf3	ENSMUSG00000015748	3.24536738	< 1e-16
Fbxo7	ENSMUSG00000001786	3.14029504	< 1e-16
Zbtb4	ENSMUSG00000018750	2.97731991	< 1e-16
Nfyb	ENSMUSG00000020248	2.81277516	< 1e-16
Kif11	ENSMUSG00000012443	2.73714726	< 1e-16
Plscr3	ENSMUSG00000019461	2.72392972	< 1e-16
Ilf2	ENSMUSG00000001016	2.41570369	< 1e-16
Setdb1	ENSMUSG00000015697	2.39216144	< 1e-16
Frs2	ENSMUSG00000020170	2.3423951	< 1e-16
Cav2	ENSMUSG00000000058	2.16089064	< 1e-16
Nbr1	ENSMUSG00000017119	2.0405694	< 1e-16
Stx5a	ENSMUSG00000010110	2.02519278	< 1e-16
Dnajc7	ENSMUSG00000014195	1.99790247	< 1e-16
Cdh1	ENSMUSG00000000303	-3.2636552	< 1e-16
Fosb	ENSMUSG00000003545	-5.3444481	< 1e-16
Tns3	ENSMUSG00000020422	-6.1990332	< 1e-16

Disease Models & Mechanisms • Supplementary information

Table S4. Top 100 differentially expressed genes in UN-SCC680 cells vs lung airway basal cells.

Name	Gene	Log2 fold change	Adj p-value
Arnt2	ENSMUSG00000015709	11.0302969	< 1e-16
Relt	ENSMUSG00000008318	10.1649645	< 1e-16
Mb	ENSMUSG00000018893	10.1322857	< 1e-16
Tmod4	ENSMUSG00000005628	9.59922685	< 1e-16
2210416O15Rik	ENSMUSG00000018378	9.37394881	< 1e-16
Serpinf1	ENSMUSG00000000753	9.32889043	< 1e-16
Dnah1	ENSMUSG00000019027	8.61534315	< 1e-16
Eno2	ENSMUSG00000004267	8.57805191	< 1e-16
Angptl4	ENSMUSG00000002289	8.48532671	< 1e-16
Hsd17b1	ENSMUSG00000019301	8.32764893	< 1e-16
Dhdh	ENSMUSG00000011382	8.28966448	< 1e-16
Msh5	ENSMUSG00000007035	7.77264297	< 1e-16
Pkib	ENSMUSG00000019876	7.49235009	< 1e-16
Atp1a2	ENSMUSG00000007097	7.42149922	< 1e-16
Hmg20b	ENSMUSG00000020232	7.16859666	< 1e-16
Vwa7	ENSMUSG00000007030	7.05128056	< 1e-16
Ttc25	ENSMUSG00000006784	6.77432375	< 1e-16
Apc2	ENSMUSG00000020135	6.7453615	< 1e-16
Rundc3a	ENSMUSG00000006575	6.63601504	< 1e-16
Plekho1	ENSMUSG00000015745	6.60749288	< 1e-16
Slc25a22	ENSMUSG00000019082	6.37722265	< 1e-16
Dnah2	ENSMUSG00000005237	6.27533395	< 1e-16
Slc12a5	ENSMUSG00000017740	6.23964264	< 1e-16
Pcsk4	ENSMUSG00000020131	6.03161707	< 1e-16
Neu1	ENSMUSG00000007038	5.92053872	< 1e-16
Zfp287	ENSMUSG00000005267	5.90424706	< 1e-16
Lamb1	ENSMUSG00000002900	5.77306125	< 1e-16
Slc5a6	ENSMUSG00000006641	5.75483201	< 1e-16
Mlxipl	ENSMUSG00000005373	5.66747245	< 1e-16
Sult2b1	ENSMUSG00000003271	5.62563762	< 1e-16
Nav1	ENSMUSG00000009418	5.37677681	< 1e-16
Cyth2	ENSMUSG00000003269	5.3735453	< 1e-16
Taf6l	ENSMUSG00000003680	5.28299905	< 1e-16
Dhx34	ENSMUSG00000006019	5.28023934	< 1e-16
Armc6	ENSMUSG00000002343	5.18914627	< 1e-16
Ppfia3	ENSMUSG00000003863	5.11440697	< 1e-16
Rpa3	ENSMUSG00000012483	5.03981044	< 1e-16
Fam184b	ENSMUSG00000015879	5.03063733	< 1e-16
Nkiras2	ENSMUSG00000017837	4.93744451	< 1e-16
Fblim1	ENSMUSG00000006219	4.81604836	< 1e-16

Yeats4	ENSMUSG00000020171	4.78608247	< 1e-16
Rasa4	ENSMUSG00000004952	4.73939553	< 1e-16
Dyrk3	ENSMUSG00000016526	4.70720213	< 1e-16
Ikbkg	ENSMUSG00000004221	4.67044294	< 1e-16
Capza2	ENSMUSG00000015733	4.65097524	< 1e-16
Snrpd1	ENSMUSG00000002477	4.59750605	< 1e-16
Dennd2c	ENSMUSG00000007379	4.56693762	< 1e-16
Zfp775	ENSMUSG00000007216	4.54053775	< 1e-16
Atp5g1	ENSMUSG00000006057	4.47354414	< 1e-16
Evi5l	ENSMUSG00000011832	4.47250718	< 1e-16
Slc7a7	ENSMUSG00000000958	4.43246762	< 1e-16
G3bp1	ENSMUSG00000018583	4.43117705	< 1e-16
Snd1	ENSMUSG00000001424	4.42161166	< 1e-16
Etv5	ENSMUSG00000013089	4.25770567	< 1e-16
Zfp385a	ENSMUSG00000000552	4.22455284	< 1e-16
Smo	ENSMUSG00000001761	4.20194912	< 1e-16
Tpra1	ENSMUSG00000002871	4.16481083	< 1e-16
Sf3a2	ENSMUSG00000020211	4.14148478	< 1e-16
Zfp959	ENSMUSG00000003198	4.12972949	< 1e-16
Rufy1	ENSMUSG00000020375	4.10957995	< 1e-16
Impdh1	ENSMUSG00000003500	4.08982027	< 1e-16
Cacnb3	ENSMUSG00000003352	4.0882497	< 1e-16
Tnk1	ENSMUSG00000001583	4.03192866	< 1e-16
Atad5	ENSMUSG00000017550	4.01868583	< 1e-16
Map1s	ENSMUSG00000019261	3.97883418	< 1e-16
Mrpl4	ENSMUSG00000003299	3.96401943	< 1e-16
Pou6f1	ENSMUSG00000009739	3.9113862	< 1e-16
Csf1	ENSMUSG00000014599	3.90900515	< 1e-16
Ptpn6	ENSMUSG00000004266	3.88326407	< 1e-16
Gtf2h4	ENSMUSG00000001524	3.76191354	< 1e-16
Rbsn	ENSMUSG00000014550	3.69277506	< 1e-16
Vps50	ENSMUSG00000001376	3.6467452	< 1e-16
Mri1	ENSMUSG00000004996	3.64571981	< 1e-16
Lmf1	ENSMUSG00000002279	3.58795081	< 1e-16
Tnpo3	ENSMUSG00000012535	3.47925466	< 1e-16
Kif11	ENSMUSG00000012443	3.46765372	< 1e-16
Cacfd1	ENSMUSG00000015488	3.45613541	< 1e-16
Nipsnap3b	ENSMUSG00000015247	3.32030006	< 1e-16
Mpnd	ENSMUSG00000003199	3.24387873	< 1e-16
Dgke	ENSMUSG00000000276	3.21013686	< 1e-16
Txn2	ENSMUSG00000005354	3.19825826	< 1e-16
Nt5c3b	ENSMUSG00000017176	3.19511096	< 1e-16
Prpf3	ENSMUSG00000015748	3.17312406	< 1e-16
Gtf2ird2	ENSMUSG00000015942	3.08465252	< 1e-16

Nek8	ENSMUSG00000017405	3.050474	< 1e-16
Cav1	ENSMUSG00000007655	3.05045233	< 1e-16
Dhx40	ENSMUSG00000018425	2.95983006	< 1e-16
Map3k11	ENSMUSG00000004054	2.94014148	< 1e-16
Anxa6	ENSMUSG00000018340	2.89648197	< 1e-16
Lnx2	ENSMUSG00000016520	2.87400655	< 1e-16
Zbtb4	ENSMUSG00000018750	2.75261519	< 1e-16
Akt2	ENSMUSG00000004056	2.70457662	< 1e-16
Stx5a	ENSMUSG00000010110	2.6825902	< 1e-16
Plscr3	ENSMUSG00000019461	2.66940471	< 1e-16
Mrpl52	ENSMUSG00000010406	2.59427956	< 1e-16
Dnajc7	ENSMUSG00000014195	2.59160738	< 1e-16
Met	ENSMUSG00000009376	2.40785736	< 1e-16
Nbr1	ENSMUSG00000017119	2.39986148	< 1e-16
Celsr1	ENSMUSG00000016028	-4.8876911	< 1e-16
Tns3	ENSMUSG00000020422	-6.4015795	< 1e-16

Disease Models & Mechanisms • Supplementary information

Table S5. Differentially expressed genes shared and non-shared between UN-SCC679 and UN-SCC680 cell lines.

UN-SCC679 (exclusive)	UN-SCC679 and UN-SCC680 (shared)	UN-SCC680 (exclusive)
Ankrd13d	*Acy3*	*Lmf1*
Atp6v1c1	*Alyref*	*Mpnd*
Cav2	*Angptl4*	*Map3k11*
Ccar1	*Ank*	*Akt2*
Cdh1	*Anxa6*	*Ikbkg*
Chat	*Apc2*	*Mri1*
Chrd	*Armc6*	*Txn2*
Dbf4	*Arnt2*	*Zfp775*
Dnah12	*Asic1*	*Pou6f1*
Fbxo7	*Aspscr1*	*Mrpl52*
Fgd6	*Atad5*	*Rpa3*
Fosb	*Atl1*	*Nipsnap3b*
Frs2	*Atp1a2*	*Celsr1*
Galc	*Atp5g1*	*Nt5c3b*
Ilf2	*Bop1*	*Nek8*
Iws1	*Brix1*	*Hsd17b1*
Matn2	*Cacfd1*	*Polk*
Mocs2	*Cacna1g*	*Carmil3*
Nfyb	*Cacnb3*	*Xpnpep3*
Npr3	*Cant1*	*Oplah*
Nudt5	*Capza2*	*Lmbr1l*
Nup155	*Casp12*	*Cox14*
Pa2g4	*Cav1*	*Map3k12*
Ppif	*Cbr2*	*Npff*
Sema5a	*Ccny*	*Nagpa*
Setdb1	*Cct5*	*Bsg*
Slc25a13	*Cenpp*	*Adamts10*
Slc5a8	*Csad*	*Incenp*
Stk3	*Csf1*	*Tmem216*
Sumf2	*Cul2*	*Pola2*
Tedc2	*Cyth2*	*Hells*
Tmbim6	*Dennd2c*	*Casp1*
Wac	*Dgke*	*Aasdhppt*
	Dhdh	
	Dhx34	
	Dhx40	
	Dlg4	

Disease Models & Mechanisms • Supplementary information

	Dnah1	
	Dnah2	
	Dnajc18	
	Dnajc7	
	Drosha	
	Dusp1	
	Dyrk3	
	Ecd	
	Ehbp1l1	
	Eif3a	
	Eno2	
	Epas1	
	Esyt1	
	Etv5	
	Evi5l	
	Faim2	
	Fam118a	
	Fam184b	
	Fam193b	
	Fblim1	
	Fech	
	Fermt3	
	G3bp1	
	Galr2	
	Gkap1	
	Gnb3	
	Gpd1	
	Gtf2h4	
	Gtf2ird2	
	Hmg20b	
	Huwe1	
	Igf2r	
	Impdh1	
	Itgb3	
	Itgb4	
	Itsn1	
	Kcnk7	
	Kif11	
	Lamb1	
	Laptm4a	
	Lnx2	
	Ltbp3	
	Map1s	
	Mb	

Disease Models & Mechanisms • Supplementary information

	Met	
	Mfsd8	
	Mib1	
	Mlxipl	
	Mms19	
	Mrpl38	
	Mrpl4	
	Msh2	
	Msh5	
	Mtdh	
	Myo1a	
	Myo1f	
	Myo1g	
	Myom1	
	Naa40	
	Nadk2	
	Nav1	
	Nbr1	
	Ndufa5	
	Neu1	
	Nkiras2	
	Nptxr	
	P3h3	
	Pabpc1	
	Pcsk4	
	Pitpnm1	
	Pkib	
	Plekho1	
	Plscr3	
	Polr2h	
	Pop1	
	Ppfia3	
	Prpf3	
	Ptpn6	
	Rad1	
	Rai14	
	Rasa4	
	Rbsn	
	Relt	
	Retreg1	
	Rfc2	
	Rufy1	
	Rundc3a	
	Samd8	

Disease Models & Mechanisms • Supplementary information

	Sdc2	
	Sema4g	
	Serpinf1	
	Sf3a2	
	Slc12a5	
	Slc25a22	
	Slc4a9	
	Slc5a6	
	Slc7a7	
	Smo	
	Snd1	
	Snrpd1	
	Stx5a	
	Sub1	
	Sult2b1	
	Taf6l	
	Tarbp2	
	Tars	
	Thoc1	
	Tmem44	
	Tmod4	
	Tnk1	
	Tnpo3	
	Tns3	
	Tpra1	
	Trio	
	Ttc25	
	Vps50	
	Vwa7	
	Xrcc6	
	Yeats4	
	Ywhaz	
	Zbtb4	
	Zdhhc6	
	Zeb1	
	Zfp287	
	Zfp385a	
	Zfp523	
	Zfp959	
	Zfr	

Disease Models & Mechanisms • Supplementary information
